# Strain‐Adaptive Liquid Metal Interfaces Overcome Poisson's Ratio Constraints in Piezoresistive Sensors for Infant Sleep Monitoring

**DOI:** 10.1002/advs.202515117

**Published:** 2025-09-14

**Authors:** Yuxiao Zhang, Chenchen Wang, Juan Tao, Shaoyu Ma, Siyu Xie, Weiwei Guo, Rongrong Bao, Jianbei Qiu, Yue Liu, Zhengwen Yang, Caofeng Pan

**Affiliations:** ^1^ College of Materials Science and Engineering Kunming University of Science and Technology Kunming 650093 P. R. China; ^2^ Institute of Atomic Manufacturing Beihang University Beijing 100191 P. R. China; ^3^ International Institute for Interdisciplinary and Frontiers Beihang University Beijing 100191 P. R. China; ^4^ Southwest United Graduate School Kunming 650093 P. R. China; ^5^ Jiashan Fudan Institute Zhejiang 314100 P. R. China; ^6^ Henan Vocational University of Science and Technology Henan 466000 P. R. China

**Keywords:** liquid metal, piezoresistive sensors, Poisson's ratio, sleep monitoring

## Abstract

Conventional porous piezoresistive sensors suffer from lateral expansion due to a positive Poisson's ratio, causing conductive network fracture and unreliable signals. Existing structural solutions are limited by high costs and poor durability. This study introduces a dynamic conductive interface mechanism using liquid metal (LM) ink to bypass Poisson's ratio limitations. By coating eutectic gallium‐indium (EGaIn) onto a hydrophilic porous thermoplastic polyurethane (TPU) scaffold, a strain‐adaptive conductive layer is constructed, where LM droplets directionally flow to fill microcracks during deformation. This mechanism retains 98.73% of initial conductive pathways under 98% tensile strain, achieving ultra‐high sensitivity (693.65 kPa^−1^, 0.32‐10.24 kPa). The LM‐based sensor demonstrates intrinsic antibacterial properties and launderability. Integrated into an intelligent infant pillow with a 16‐chanels sensor array, the system enables real‐time cephalic pressure monitoring and edge‐computed posture correction via a companion app. This work proposes a material‐mechanics co‐design strategy to overcome Poisson ratio constraints, advancing high‐performance, scalable wearable biomedical devices.

## Graphical abstract

This work overcomes Poisson's ratio limitations in piezoresistive sensors via strain‐adaptive liquid metal (LM) interfaces. EGaIn‐coated TPU scaffolds enable directional LM flow to fill microcracks under deformation, achieving ultra‐high sensitivity (693.65 kPa^-1^). Demonstrated in a 16‐channel infant pillow system for real‐time cephalic pressure monitoring and posture correction, featuring intrinsic antibacterial properties and mechanical robustness after repeated washing.

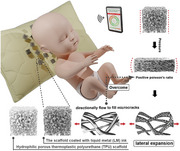

## 1 Introduction

Flexible piezoresistive sensors have been extensively explored for healthcare monitoring and human‐machine interaction.^[^ ^1^ ^]^ Porous‐structured piezoresistive sensors, owing to their exceptional compressibility and strain amplification effects, have emerged as one of the predominant strategies for achieving high‐sensitivity pressure detection. (Sensitivity is defined as the ratio of the change in the sensor's output signal to the change in input pressure, with units of kPa^−1^) In recent years, researchers have developed high‐performance sensors by constructing polymer porous scaffolds via the freeze‐drying method, phase inversion‐sacrificial templating method, 3D printing technology,^[^ ^2^ ^]^ and incorporating conductive fillers such as carbon nanotubes (CNTs), graphene.^[^ ^3^ ^]^ However, under longitudinal compression, the positive Poisson's ratio (ν = 0.3–0.5) of the polymer porous scaffold induces lateral expansion, leading to stretching‐induced fracture of the conductive network. This results in hysteresis in resistance response or even signal inversion, severely compromising measurement reliability. Significantly, it is a common phenomenon that the positive Poisson's ratio of ordinary porous structures induces lateral expansion during vertical compression, which causes irreversible cracking of conductive networks and non‐monotonic resistance response. This intrinsic conflict between mechanical deformation and electrical stability remains a critical obstacle for high‐performance sensing.

To mitigate the Poisson's ratio effect, researchers worldwide have proposed various structural design strategies. For instance, through 3D printing or mechanical metamaterial design (e.g., rotating square units and re‐entrant honeycomb structures), bidirectional contraction/expansion is generated under compression/tension, thereby enhancing conductive pathways or piezoelectric effects.^[^ ^4^ ^]^ Similarly, by integrating positive and negative Poisson's ratio elements, constant lateral dimensions are achieved to eliminate cross‐axis interference, thereby enabling decoupled detection of dual‐axis stimuli.^[^ ^4^, ^5^ ^]^ By leveraging a meta‐crack structure combined with a negative Poisson's ratio substrate to facilitate rapid crack opening, the strain response is significantly enhanced, enabling high sensitivity even under minimal strain.^[^ ^6^ ^]^ Constructing porous structures through bidirectional freeze‐drying or chemical crosslinking (e.g., polyethylene glycol (PEG) and incorporating Negative Poisson's Ratio characteristics to enhance pressure/strain responsiveness.^[^ ^4^, ^7^ ^]^ However, these approaches rely on intricate molds or directional freeze‐casting processes, resulting in prohibitively high fabrication costs and scalability challenges. Furthermore, hierarchically porous structures exhibit insufficient mechanical robustness, prone to structural collapse under prolonged cyclic loading. Consequently, circumventing Poisson's ratio limitations through material innovation rather than complex structural engineering has emerged as a critical yet unresolved challenge in this field.

This study proposes a novel dynamic conductive interface mechanism based on LM ink, which circumvents the strain‐sensitive limitations of conventional solid conductive materials. By modifying and coating eutectic EGaIn onto the surface of a hydrophilic porous TPU skeleton, we constructed a strain‐adaptive conductive layer that effectively suppresses contact degradation caused by positive Poisson's ratio effects. Experimental and simulation results demonstrate that LM droplets undergo strain‐driven directional flow under deformation, filling microcracks generated by Poisson expansion. This mechanism maintains 98.73% of the initial conductive pathway density even under 98% tensile strain. The fabricated sensor achieves an ultra‐high sensitivity of 693.65 kPa^−1^ (0.32–10.24 kPa), outperforming solid conductive materials systems in ΔI/I_0_ improvement. Our work provides a material‐mechanics co‐design strategy to overcome Poisson ratio‐induced physical constraints in flexible electronics. Furthermore, the intrinsic antibacterial properties of LM yield a maximum inhibition zone diameter of 7 mm against Staphylococcus aureus, while the sensor retains exceptional performance after 5 high‐power ultrasonic washing cycles. Integrated into an intelligent infant pillow, the optimized 4 × 4 sensor array enables real‐time monitoring of cephalic pressure distribution. The embedded system automatically identifies abnormal head positions via edge computing algorithms and delivers corrective suggestions via a companion app. This breakthrough establishes a way for rare metal‐based functional materials in next‐generation wearable biomedical devices.

## 2 Results and Discussion

### 2.1 Structure Design and Principle

**Figure 1:**
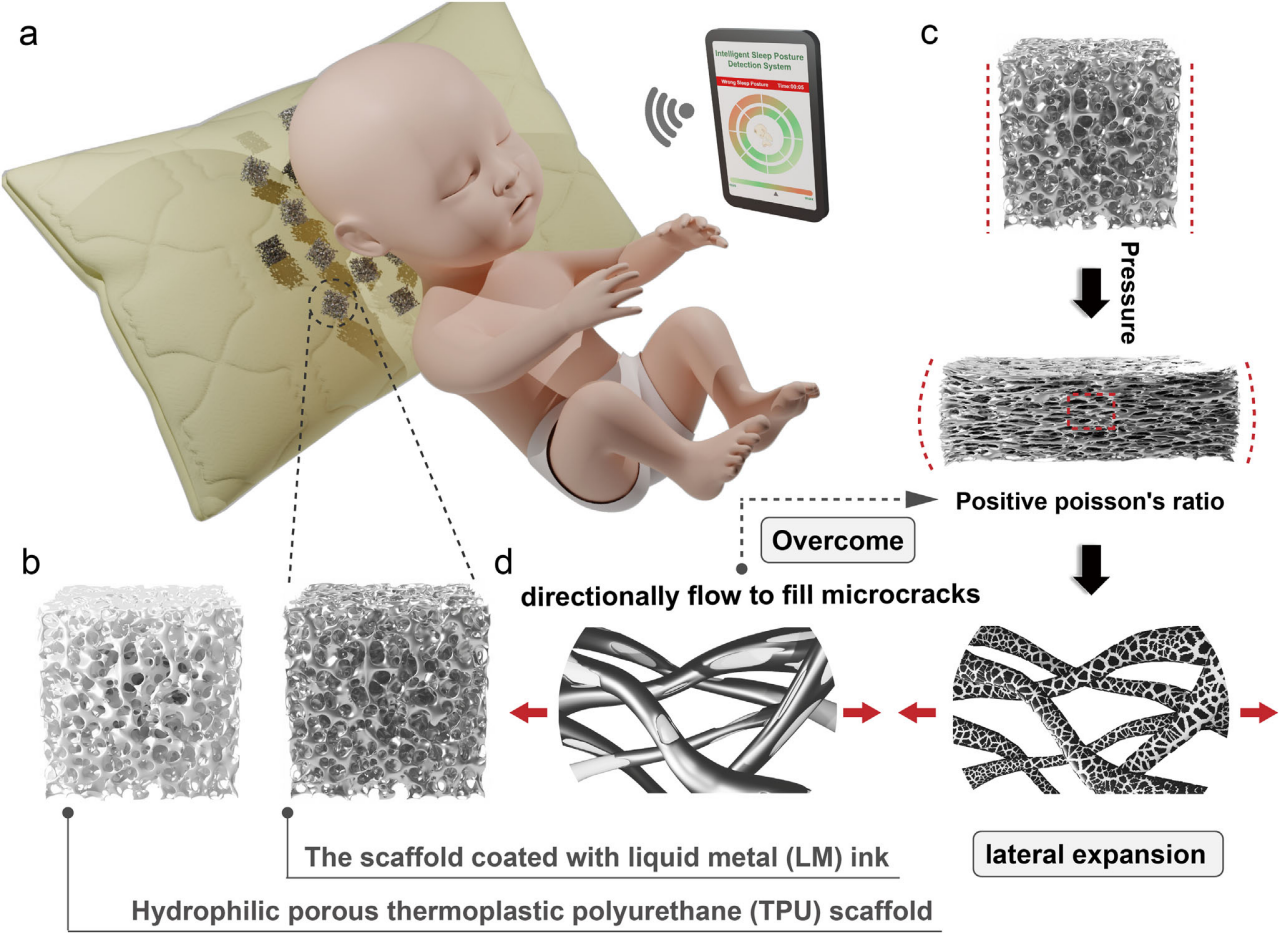
a) Integrated infant sleep monitoring system. b) Pressure‐sensitive unit structure: Internal TPU scaffold and LM ink‐coated TPU scaffold. c) Positive Poisson's ratio effect exhibited by the TPU scaffold under compression. d) Schematic illustration of lateral tensile deformation in conventional solid conductive materials and LM under compression. The LM avoids transverse brittle fracture after lateral stretching through capillary‐driven droplet flow.

The infant's skull is soft and pliable, particularly during the first six months. Prolonged fixed sleeping positions can exert sustained pressure on specific areas of the head, leading to skull deformation. Pressure monitoring technology provides real‐time feedback on pressure points, alerting caregivers to adjust the infant's head position and prevent excessive localized pressure. **Figure** **1**a demonstrates an integrated pressure‐sensing pillow designed to monitor pressure distribution across an infant's head contact surface during sleep, as well as track sleeping postures. The embedded system utilizes edge computing algorithms to automatically detect abnormal head positions, with data synchronized in real time to parental devices for dynamic alerts. This intelligent sleep monitoring system demonstrates exceptional performance through the synergy of structural design and functional materials.

As shown in Figure 1b, the pressure‐sensitive pillow employs a porous architecture consisting of a TPU skeleton with eutectic EgaIn onto the surface. The open‐cell TPU skeleton consists of interconnected struts.^[^ ^8^ ^]^ Under compression, the struts undergo bending rather than direct compression. When compressed vertically, the bending of struts causes the skeleton to expand laterally, leading to transverse expansion. Additionally, TPU is composed of alternating soft and hard segments.^[^ ^9^ ^]^ Under compression, the soft segment molecular chains extend and align orientationally, while the hard segments act as physical crosslinking points to restrict chain slippage. This constrained molecular motion dissipates energy through lateral deformation rather than purely longitudinal compression. Consequently, the positive Poisson's ratio effect in TPU porous scaffolds arises from the combined influence of their pore morphology and skeleton topology.^[^ ^10^ ^]^ Moreover, this positive Poisson's ratio effect can result in more uniform pressure distribution across the sensing layer, reducing stress concentration and preventing localized overload, thereby making it suitable for infant head pressure monitoring. However, the lateral expansion of the sensor under longitudinal compression may cause stretching‐induced rupture of the conductive network, leading to hysteresis in resistance response or even signal inversion, which severely compromises measurement reliability (Figure 1c). Additionally, lateral expansion can drive the material into a nonlinear elastic region, resulting in deviation from the linear relationship between sensing signals and applied pressure. Hence, the dynamic conductive interface mechanism of LM effectively suppresses contact degradation in conventional solid conductive materials caused by positive Poisson's ratio effects, as its solid‐liquid phase transition behavior enables self‐healing reorganization of the conductive network through viscoelastic flow during lateral stretching.

Schematic diagrams illustrating the lateral tensile deformation of conventional solid conductive materials and LM under compression are presented in Figure 1d. Conventional solid conductive materials, such as graphite/CNTs composites, exhibit brittle fracture under lateral stretching. The interparticle distance linearly increases with matrix deformation, leading to an exponential decrease in electron tunneling probability. Upon unloading, plastic deformation and contact point separation result in a permanent resistance increase. In contrast, LM undergoes shear‐thinning under strain, where capillary action fills microcracks. Droplets form chain‐like structures along the stretching direction, maintaining connectivity above the percolation threshold. Rupture of the oxide layer releases fresh LM, enabling rapid restoration of electrical conductivity. Thus, a strain‐adaptive conductive layer that effectively suppresses contact degradation caused by positive Poisson's ratio effects by modifying and coating eutectic EGaIn onto the surface of a hydrophilic porous TPU skeleton, which can significantly enhance the sensitivity of the pressure sensor.

### 2.2 Modification and Characterization of TPU Porous Scaffolds

In the process of compounding TPU with LM, strengthening interfacial interactions can be achieved through polarity matching. TPU typically exhibits hydrophobicity due to the microphase separation between its soft segments (e.g., polyester/polyether) and hard segments (urethane groups), and the unmodified hydrophobic TPU, characterized by low surface energy, leads to poor interfacial wettability, uneven dispersion of LM, and weak interfacial bonding. Introducing hydroxyl or ether groups from PEG significantly enhances the surface polarity of TPU, enabling stable bonding with the LM via hydrogen bonds or coordination interactions.^[^ ^9^, ^10^, ^11^ ^]^ This improves the spreading and dispersion of the LM within the matrix while reducing interfacial defects. Simultaneously, the hydrophilic interface can buffer dynamic stress, preventing interfacial slippage of the LM during deformation and avoiding disruption of conductive pathways.

**Figure 2:**
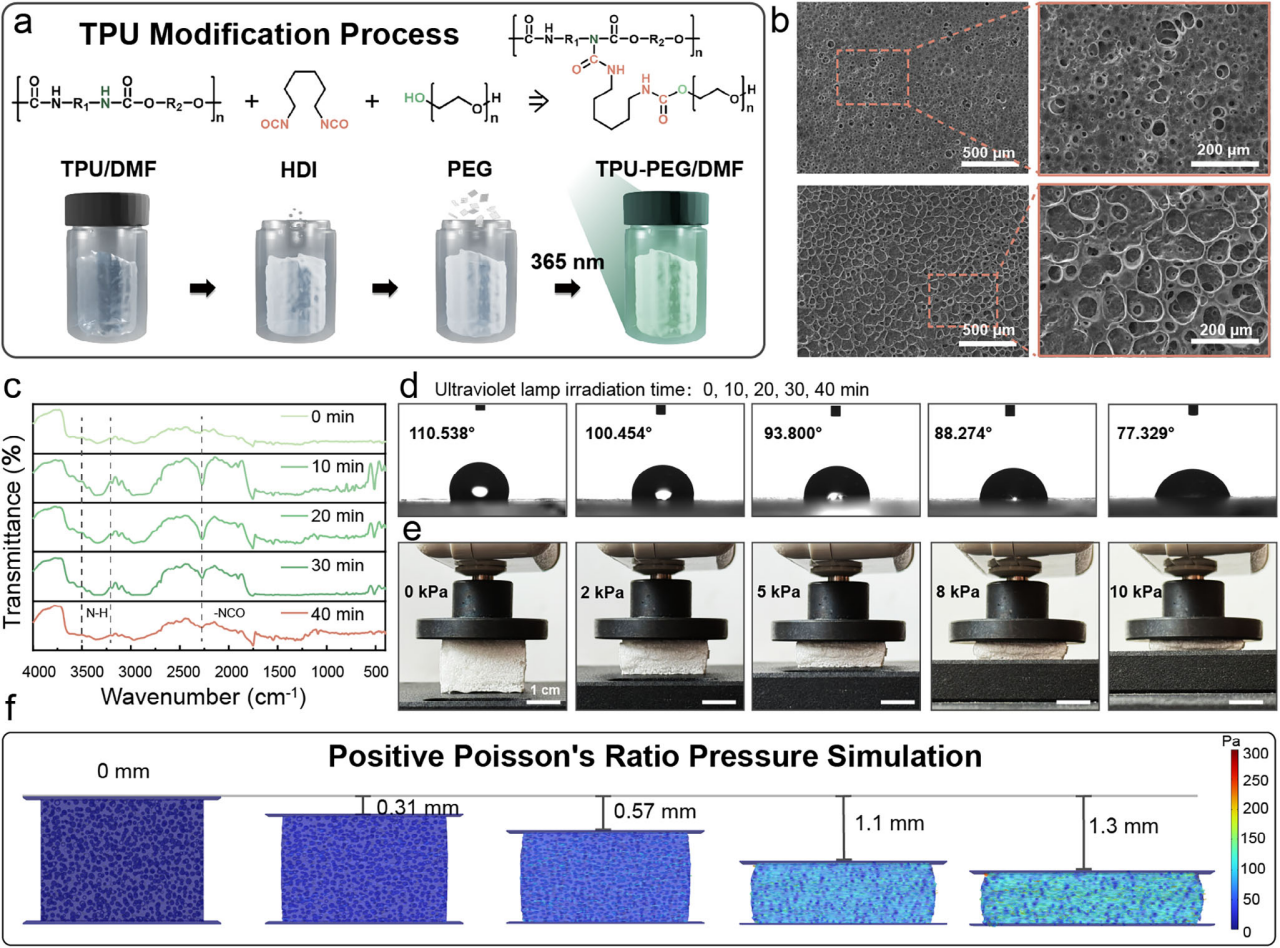
a) Modification process of TPU and chemical structures of the products. b) Surface morphology comparison between unmodified TPU and modified TPU. (Accelerating voltage: 20 kV) c) FTIR spectra at different UV irradiation durations. (Spectral resolution: 4 cm^−1^, number of scans:16) d) Water contact angles before and after modification. e) Pressure‐sensitive unit under different compression states. f) Finite element simulation of the pressure‐sensitive unit under compression.

The modification process of TPU is illustrated in **Figure** **2**a. The TPU has been pretreated with methanol for 24 h to remove surface small‐molecule residues. Subsequently, under a nitrogen atmosphere, TPU has been reacted with 1,6‐Diisocyanatohexane (HDI) in the presence of 0.2 wt% dibutyltin dilaurate (DBTDL) as a catalyst in a solvent for a period of time to obtain isocyanate‐grafted TPU (TPU‐HDI). Finally, TPU‐HDI has been reacted with PEG at 35 °C under nitrogen for 24 h to yield TPU‐PEG. Figure 2b shows the SEM images of modified and unmodified TPU films prepared by the phase inversion method. The comparative analysis reveals that the PEG/HDI modification results in more distinct alternating hydrophilic and hydrophobic domains in the TPU films, analogous to a sponge‐like porous morphology. This phenomenon arises because the soft segments of conventional TPU exhibit strong hydrophobicity, while the hard segments possess limited polarity, leading to weak microphase separation. By grafting PEG, a highly hydrophilic soft segment rich in ether linkages (─O─) and hydroxyl groups (─OH), onto the TPU chains, the polarity of the soft segments is significantly enhanced. The increased polarity contrast between the hydrophilic PEG‐modified soft segments and the hydrophobic urethane‐based hard segments thermodynamically favors phase separation. Meanwhile, HDI acts as a crosslinking agent, reacting via its isocyanate groups (─NCO) with hydroxyl or amino groups in TPU/PEG to form a 3D crosslinked network. This network hinders the dynamic mixing of soft and hard segments, compelling the two phases to separate earlier and more thoroughly during the film‐forming process. Under UV irradiation, reaction durations of 10, 20, 30, and 40 min have been investigated to optimize the grafting of HDI and PEG onto TPU. Figure 2c demonstrates that the ─NCO characteristic peak at 2272 cm^−1^ gradually diminishes with prolonged reaction time, while the absorption bands at 3290–3376 cm^−1^ evolve from sharp, narrow peaks to broadened features, confirming the effective PEG grafting. Furthermore, the contact angles of modified TPU films with different grafting reaction times have been tested. All test droplets maintained a uniform volume of 10 µL, with a surface equilibration time of 10 s. Water droplets exhibit a large contact angle of 110.538° on unmodified TPU films. As the reaction time increased, the contact angle progressively decreased to 77.329°, indicating a significant enhancement in wettability in Figure 2d. Thus, 40 min was selected as the optimal TPU‐PEG grafting duration to ensure scaffold hydrophilicity.

The modified TPU has been fabricated into a porous scaffold for pressure sensor applications via a phase inversion method. The scaffold exhibited a 3D interconnected porous architecture with a pore diameter of 10–40 µm and porosity of 45.5%, as characterized by SEM analysis in Figure S1 (Supporting Information). During uniaxial compression testing (0–10 kPa, 0.2 s^−1^ strain rate), the porous TPU scaffold demonstrated a distinctive positive Poisson's ratio (ν = +0.185), where transverse expansion accompanied axial compression in Figure 2e. Lateral and longitudinal strain values under normal pressure are supplemented in Figure S2 (Supporting Information). The calculation formula for Poisson's ratio is given in Equation S1 (Supporting Information). This anomalous behavior originated from the synergistic effect of pore wall buckling and strut reorientation in the hierarchical porous structure. Finite element analysis (FEA) with explicit pore geometry reconstruction in Figure 2f. This COMSOL model measures 2 mm in length, 0.2 mm in width, and 1.5 mm in height, with pore diameters ranging from 20 to 40 µm and a porosity of 40% (Figure S3, Supporting Information). The simulated Poisson's ratio evolution (ν_simulation = +0.192) aligned within 3.78% error with experimental measurements, validating the mechanical coupling mechanism between pore morphology and macroscopic deformation.

### 2.3 Modification and Characterization of LM Inks

**Figure 3:**
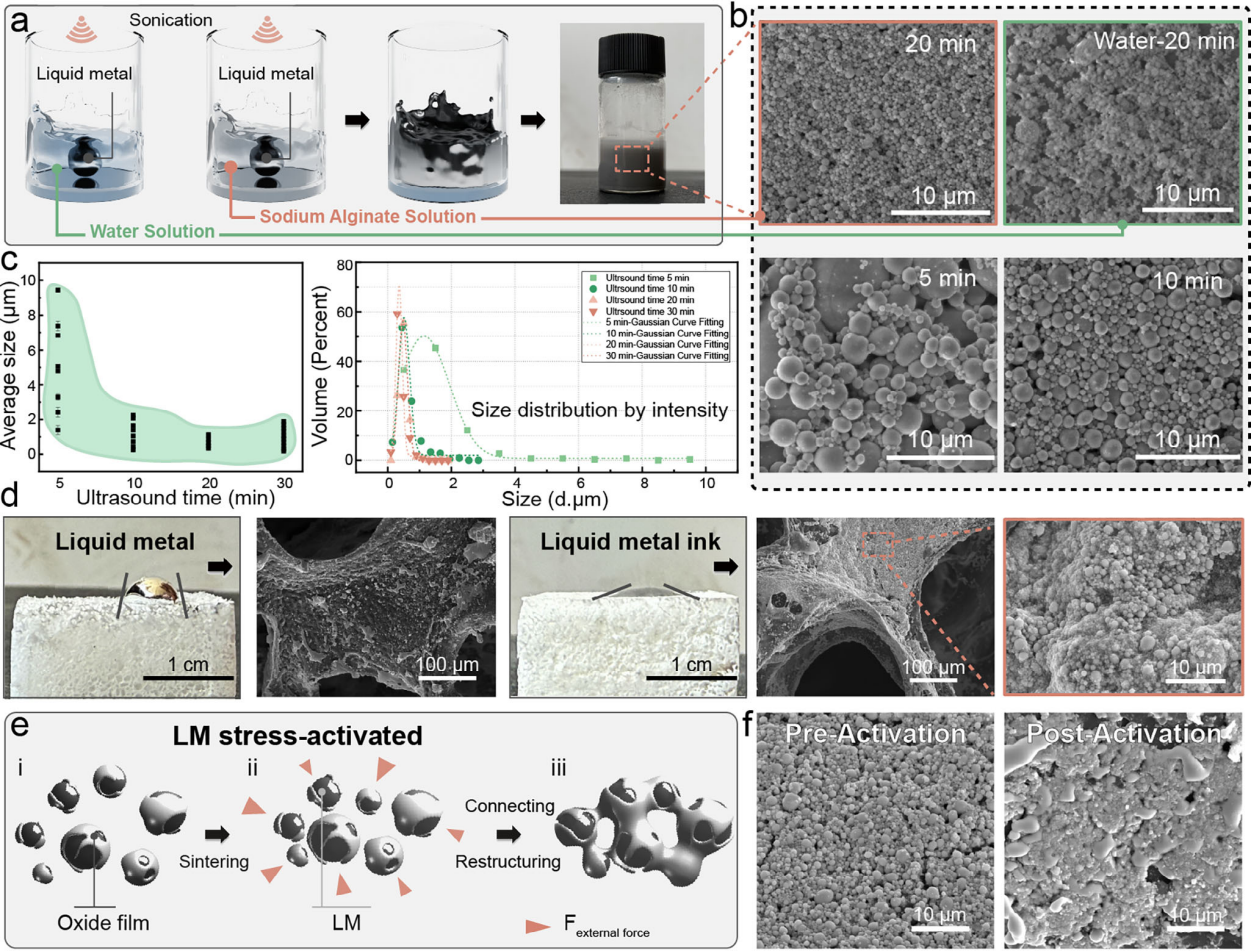
a) Specific modification measures for LM (with water as the control group). b) SEM characterization of the SA solution after 5, 10, and 20 mins ultrasonication and the aqueous solution after 20 mins ultrasonication. (Accelerating voltage: 10 kV) c) Size distribution of LM particles in SA solution. d) Contact angles (Droplet volume: 30 µL, surface equilibration time: 10 s) and surface SEM images of LM and LMs ink adhered on TPU scaffolds. (Accelerating voltage: 20 kV) e) Stress‐activation process of LM. f) SEM images of LM before and after stress‐activation. (Accelerating voltage: 20 kV).

In addition to the hydrophilic modification of TPU, LMs tend to aggregate due to their high surface tension, resulting in inhomogeneous dispersion within the TPU matrix and weak interfacial bonding.^[^ ^12^ ^]^ This phenomenon readily induces stress concentration and conductive network fracture. Consequently, modifying LMs into functional inks emerges as a pivotal strategy.^[^ ^13^ ^]^ Therefore, the LM has been modified through a synergistic approach combining sodium alginate (SA) and ultrasonic treatment, as illustrated in **Figure** **3**a. Under ultrasonic cavitation, the LM is fragmented into micron‐sized particles, forming a uniformly dispersed two‐phase system. Concurrently, sodium alginate molecules formed an adsorbed coating via coordination interactions between their carboxyl groups (─COOH) and the oxidized surface layer (Ga_2_O_3_) of the LM. This coating mechanism, driven by spatial steric hindrance and electrostatic repulsion effects, effectively suppressed particle reaggregation. Compared to the ultrasonic fragmentation of LM in pure water, no significant oxidation has been observed in the SA‐assisted process.

Further optimization of the ink's particle size has been systematically conducted by tailoring the ultrasonication duration, accompanied by statistical evaluations of the LM particle size distribution in Figure 3b,c. When the ultrasonication duration is short (≈5 mins), the LM particles exhibit a non‐uniform size distribution, likely due to insufficient cavitation energy to overcome the inherent surface tension. As the ultrasonication time increased (10–30 mins), the average particle size progressively decreased from an initial ≈5 to ≈500 nm, aligning with the intensified cavitation‐driven fragmentation. This refinement in particle size ensured a homogeneous dispersion critical for achieving stable conductive pathways within the TPU matrix. When the ultrasonication duration is extended to 40 mins, significant oxidation occurred, likely due to the excessively reduced particle size that increased the specific surface area and oxygen permeability, even under the protective effect of sodium alginate (Figure S4, Supporting Information).

In contrast to LM, the wetting behavior of the LM‐based ink is not restricted by high surface tension or surface oxide films, but is primarily governed by the interfacial interactions between the aqueous solution and the substrate. As shown in Figure 3d, the wettability of LM‐based inks on TPU porous scaffolds is significantly enhanced compared to pristine LM, as evidenced by optical photos. Contact angle measurements reveal that the LM ink exhibits a reduced contact angle of 46.9° on TPU surfaces (vs 64.7° for pure LM, 17.8° for SA‐stabilized LM ink), attributed to the synergistic effects of reduced surface tension by SA modification and the elimination of oxide‐mediated interfacial barriers. SEM images demonstrate that the SA‐stabilized LM ink achieved conformal coating on the TPU scaffold, with intimate interfacial contact facilitated by hydrogen bonding between SA hydroxyl groups and TPU urethane linkages. This optimized wetting behavior ensured the formation of percolated conductive networks critical for high‐performance pressure sensors. However, the presence of oxide layers on the surface of LM particles impedes direct interparticle contact, resulting in low initial conductivity. Therefore, a stress‐activation process is essential for the as‐prepared LM‐based ink to disrupt the oxide barriers. As illustrated in Figure 3e, external stress is applied to induce deformation of LM particles, where the brittle oxide layer (Ga_2_O_3_) undergoes strain mismatch and generates microcracks. This mechanical perturbation enables the release of the LM core from the fractured regions, exposing fresh metallic surfaces and facilitating metal‐to‐metal contact, thereby establishing continuous conductive pathways. SEM images in Figure 3f further reveal that under stress activation, the ruptured oxide layer allows the LM ink to bridge adjacent particles and infiltrate the micropores of the TPU, which synergistically enhances interfacial adhesion and electrical stability.

### 2.4 Performance Characterization of Sensors

**Figure 4:**
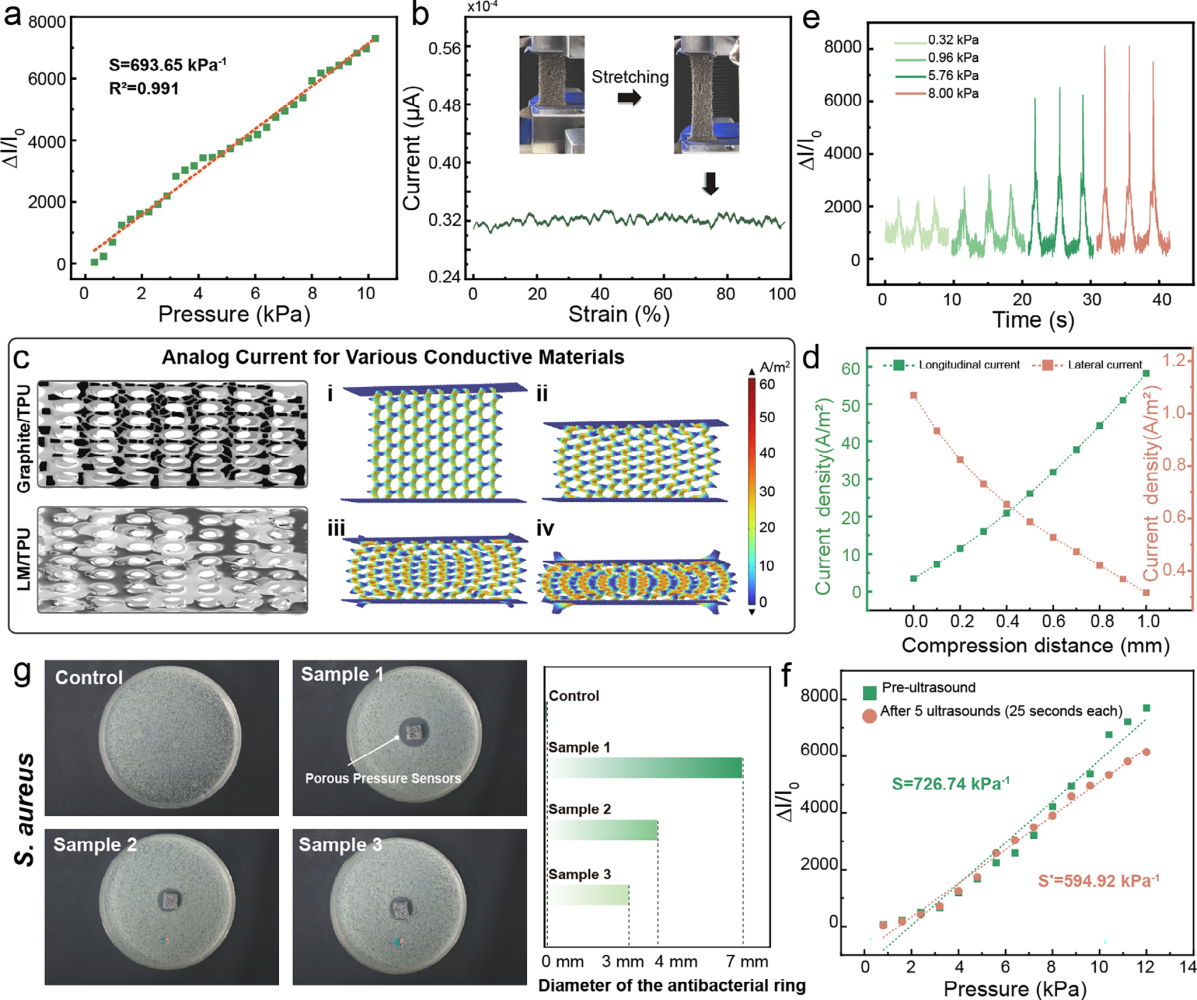
a) Sensitivity of the sensor unit under axial pressure. b) Current fluctuation of the sensor unit under transverse tensile strain. c,d) Simulation of the sensor unit via coupled Electric Currents and Solid Mechanics physics interfaces and demonstration of simulation results. e) Dynamic response of the sensor unit. f) Sensitivity comparison of the sensor unit after five consecutive ultrasonic cleaning cycles. g) Antibacterial testing of the sensor unit (test strain: Staphylococcus aureus ATCC 6538).

To verify the influence of the porous sensing layer's relaxation behavior under dynamic loading on macroscopic mechanical response, mechanical property testing was conducted prior to evaluating the device's sensing performance. Results demonstrate that the viscoelastic properties of this sensing layer exhibit significant and consistent frequency dependence within the 0.1–10 Hz range. Furthermore, the porous structure substantially reduces the overall stiffness of the material. For TPU porous materials, due to vigorous compression of internal gas under adiabatic conditions during high‐speed loading, identical external forces generate greater deformation. This gas–solid coupling effect is a distinctive phenomenon unique to porous viscoelastic materials. Detailed explanations are provided in Figures S5–S8 (Supporting Information). The pressure‐sensitivity relationship of the sensor is depicted in **Figure** **4**a, which shows an ultrahigh sensitivity of 693.65 kPa^−1^ (being a conservative estimate, the sensitivity of application‐integrated sensors exceeds this value) under the range of 0.32–10.24 kPa with a linearity R^2^ = 0.991, calculated from the slope between coordinates. Notably, the sensor demonstrates a high‐sensitivity response under axial compressive strain, while exhibiting merely 1.27% resistance fluctuation across a transverse tensile strain range of 3.7–98.0% in Figure 4b, validating that the LM‐based ink effectively suppresses the conductive pathway density attenuation caused by transverse expansion in positive Poisson's ratio materials, via strain‐adaptive conductive network interface reconstruction. Moreover, it employs COMSOL to model the piezoresistive behavior of compressed anisotropic traditional electric materials, such as graphite. A 3D geometry has been imported and simulated using coupled Electric Currents and Solid Mechanics physics interfaces. Material properties have been defined with anisotropic electrical conductivity (σ), where conductivity varies with compressive strain following σ = σ_0_/(1+ε), alongside standard elastic parameters (Young's modulus, Poisson's ratio). Custom piezoresistive coupling has been implemented by linking strain results from Solid Mechanics to the conductivity variable. A refined mesh was applied to resolve current density gradients near edges and compressed regions. (Figure 4c). Simulation results in Figure 4d indicate that the lateral current density and longitudinal current density exhibit opposing trends with increasing compression displacement. The experimental data agree with the simulation results (see Figure S9, Supporting Information). Specifically, for conventional conductive materials, the positive Poisson's ratio effect counteracts the enhancement of effective current under compression. The strain‐adaptive LM interface effectively overcomes the limitations imposed by Poisson's ratio. The interconnectivity status of this LM ink versus conventional rigid material graphite powder under applied stress is shown in Figure S10 (Supporting Information). Figure S11 (Supporting Information) simulates the strain‐flow coupling process of LM ink. The sensor exhibits highly stable response characteristics under repeated pressure applications across four distinct (0.32, 0.96, 5.76, and 8.00 kPa) pressures in Figure 4e. And it achieves a response time of 0.93 s and recovery time of 1.92 s, as measured at the 90% signal threshold under 3 kPa in Figure S12 (Supporting Information). Over 5500 compression cycles (0–8 kPa), the sensor maintains current stability (Figure S13, Supporting Information), and the minimum detection limit is 0.3 kPa (Figure S14, Supporting Information).

To validate the launderability of the device, the sensor has been subjected to high‐power ultrasonic cleaning (power density: 120 W cm^−2^, frequency: 40 kHz) for five consecutive cycles (25 s per cycle), simulating durability challenges under extreme mechanical–chemical coupling conditions in Figure 4f. The photos have been supplied in Figure S15 (Supporting Information). Post‐treatment analysis revealed a sensitivity retention rate of 81.86% (initial: 726.74 kPa^−1^, post‐treatment: 594.92 kPa^−1^), confirming the robustness of the device against high‐frequency mechanical shocks. To validate performance under real‐world laundering conditions, standardized washing machine tests have been conducted (Panasonic, rated frequency: 50 Hz, washing power: 530 W). Employing a controlled 25‐s cycle duration, the device exhibited 71.31% pressure response sensitivity retention following 21 cleaning cycles (Figure S16, Supporting Information). Table S1 (Supporting Information) lists the sensitivity and its value retention of the component corresponding to the wash cycles. The analysis of failure causes during the device washing process is provided in the supporting information, where the loss of functional materials and surface oxidation behavior are identified as contributors to the failure (as observed in Figures S17–S19, Supporting Information). To better align with practical application scenarios, antibacterial testing has been conducted in Figure 4g. Based on the inhibition zone assay (ASTM E2149 standard, Staphylococcus aureus ATCC 6538), the sample exhibits a maximum inhibition zone diameter of 7.0 ± 0.5 mm (*n* = 3), significantly exceeding the negative control group (0 mm), confirming its robust contact‐active antibacterial properties. This efficacy can be attributed to the following reasons. One is that the LM surface oxide layer (Ga_2_O_3_) undergoes gradual hydrolysis in physiological environments, releasing Ga^3^⁺ ions. These ions competitively inhibit bacterial iron metabolism, disrupting cytochrome enzyme activity and causing energy synthesis failure. Another is that during solidification, the LM interface forms nanoscale sharp structures, which mechanically puncture bacterial cell walls via localized stress concentration. By synergizing triple mechanisms (ion toxicity, physical disruption, and oxidative stress), the sensor achieves efficient eradication of S. aureus, ensuring infant environmental safety in wearable healthcare applications.

### 2.5 Infant Sleep Monitoring System

Positional Plagiocephaly Syndrome (PPS) has become a prominent issue in modern infant care, with American Academy of Pediatrics 2022 data showing an incidence rate as high as 48%. It can lead to severe consequences, including permanent cranial asymmetry, abnormal facial development, and impaired motor skills.^[^ ^14^ ^]^ Research by Argenta et al. has confirmed that persistent unilateral pressure leads to premature suture closure in the occipital region, not only causing abnormal head shape appearance but also, due to intracranial space imbalance, affecting cerebellar development and increasing the risk of sensory integration disorders. The World Health Organization explicitly identifies sleep posture management as the core measure for PPS prevention. However, existing reliance on parental visual checks suffers from three key bottlenecks. First, lack of continuity leading to blind spots in nighttime monitoring; Second, inability to quantify leading to non‐visualization of pressure distribution; Third, delayed response resulting in the scenario where “detection occurs only after substantial pressure has already formed”. Therefore, traditional methods relying on parental visual observation have significant delays, making it difficult to meet early intervention needs.^[^ ^15^ ^]^

**Figure 5:**
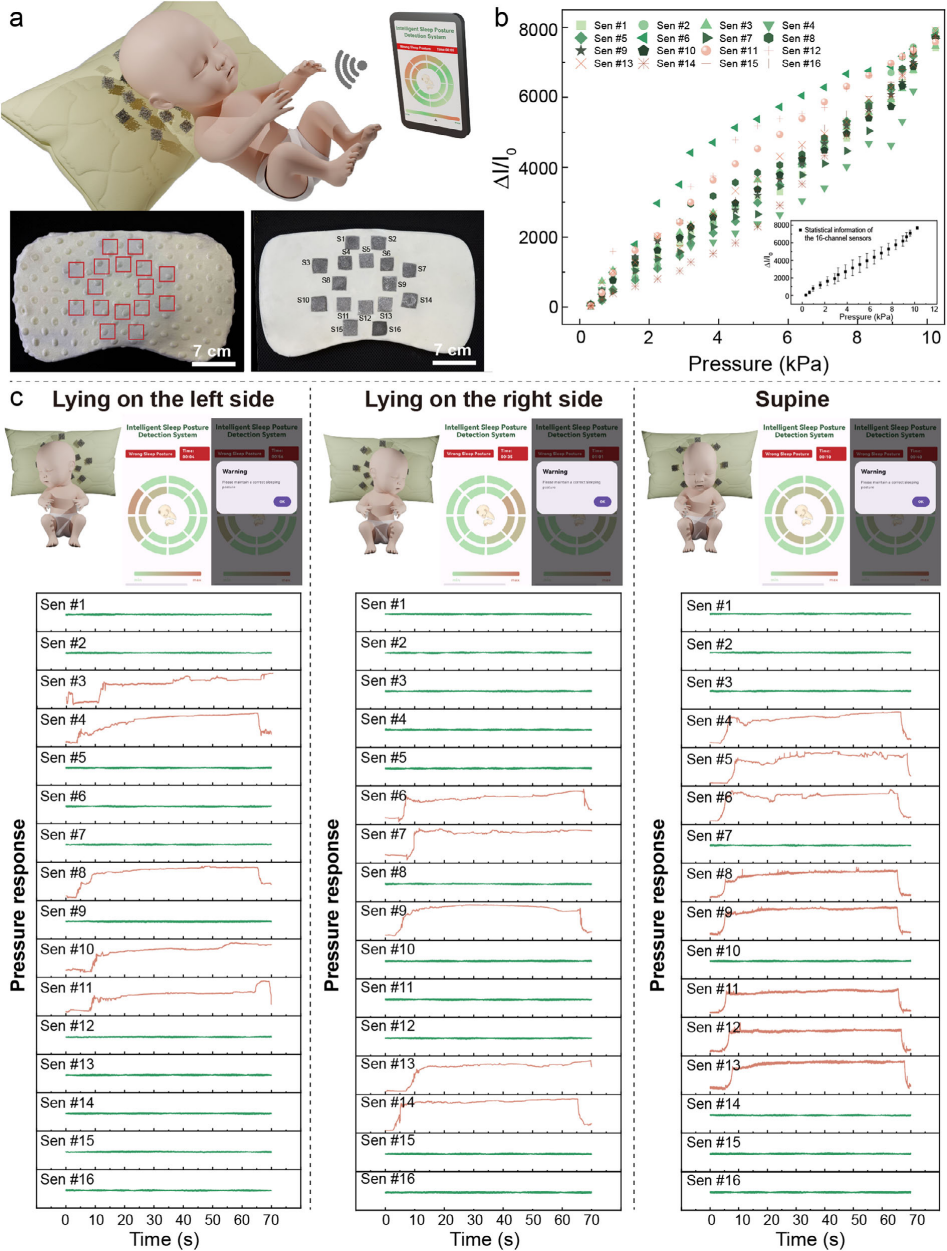
a) Integrated infant sleep monitoring system: Comprising an infant pillow embedded with a 16‐channel sensor array and a real‐time monitoring APP interface. b) Consistency validation of the 16‐channel sensor array. c) Pressure states of each sensor group during left‐lateral position, right‐lateral position, and supine position of the infant, along with pressure cloud visualization on the APP interface and pop‐up alert triggered after maintaining incorrect posture for >60 s.

To overcome this limitation, precise control over infant sleep posture dynamics is achieved by covering pressure‐sensitive areas of the occiput with a 16‐channel sensor array (**Figure** **5**a) combined with an adaptive threshold algorithm. The entire smart pillow system adopts a three‐layer modular architecture. The sensing layer employs a double‐ring distribution layout precisely set at 2.5 mm spacing based on infant occipital size, and the height is designed at 3 mm based on infant cervical spine development. The porous structure achieves an air circulation rate that effectively reduces stuffiness. The processing layer uses a 04M‐V3‐Bl chip for signal amplification and ADC conversion (Figure S20, Supporting Information). The interaction layer transmits data to mobile terminals via low‐power Bluetooth. Infant sleep posture recognition is based on the extraction of signals from dynamic pressure distribution matrices. Spatial features require the number of sensors under sustained pressure, N ≥ 4 to effectively exclude accidental limb contact. Temporal features require pressure to be maintained for t ≥ 60 s to prevent brief turning interference. Additionally, distribution features are determined by a focus index; for example, if the left focus degree D_L > 0.7, it triggers a left‐side lying alarm. The acquisition board reads the 16‐channel data in real‐time, calculates the weighted pressure values for each region, and activates the corresponding alarm protocol when the pressure distribution in a specific region continuously exceeds the threshold for 60 s.

According to medical literature, the normal range of intracranial pressure (ICP) in infants in the supine position is 1.5–6.0 mmHg, which converts to ≈0.2–0.8 kPa.^[^ ^16^ ^]^ To verify sensor consistency, their fundamental electrical performance has been tested in Figure 5b. Results show a small difference among the 16 sensors within the simulated head pressure range (0.32–10.24 kPa). The 16‐channel device exhibits exceptional consistency, evidenced by a residual sum of squares (RSS) of ≈3.51 for output currents across varying pressures and an error bar length of 0.148 times the mean value in the illustration. All channels exhibit excellent linear responses (R^2^ = 0.999), with an average sensitivity of approximately 759.02 kPa^−1^. The corresponding boxplot is provided in Figure S21 (Supporting Information). Figure 5c clearly shows the pressure status of the sensor groups when the infant is in left‐side lying, right‐side lying, and supine positions, visually verifying the relationship between pressure distribution and posture. When the infant is in left‐side lying, sensor groups S3 (Sensor 3), S4, S8, S10, and S11 sustain pressure, and when this lasts over 60 s, the APP immediately pushes an alarm signal. After parental adjustment, monitoring data shows pressure returns to a balanced distribution across regions. When the infant turns to the right‐side lying and supine positions, sensor groups S6, S7, S9, S13, and S14, and sensor groups S4, S5, S6, S8, S9, S11, S12, and S13, respectively, sustain pressure. Compared to traditional solutions, this system provides timely alarms upon detecting persistent unilateral pressure, enabling parents to intervene before cranial deformation occurs. It establishes an early warning window ahead of deformity formation and provides visualization of pressure distribution to guide precise posture adjustment.

## 3 Conclusion

This study demonstrates a material‐mechanics co‐design strategy that overcomes the limitations of conventional porous piezoresistive sensors‐including lateral expansion, conductive network fracture, and unreliable signals caused by positive Poisson's ratio – through a dynamic conductive interface mechanism utilizing LM ink. By coating eutectic EGaIn ink onto a hydrophilic porous TPU scaffold, a strain‐adaptive conductive layer is constructed. Within this layer, LM droplets directionally flow to fill microcracks generated during deformation. This unique mechanism effectively suppresses contact degradation induced by the Poisson's ratio effect. Remarkably, it retains 98.73% of the initial conductive pathway density even under extreme 98% tensile strain, achieving ultra‐high sensitivity (693.65 kPa^−1^, 0.32–10.24 kPa). This work establishes a pathway for wearable biomedical devices, as exemplified by its successful integration into an intelligent infant sleep monitoring pillow, enabling real‐time pressure mapping and posture correction.

## 4 Experimental Section

### Preparation of TPU Porous Scaffold

TPU (Wangda Plastic Raw Materials Co., Ltd.) was mixed with N,N‐dimethylformamide (DMF, 99.5% purity, analytical reagent, Aladdin Biochemical Technology Co., Ltd.) at a concentration of 30 wt%. The mixture was stirred and dissolved at 60 °C for 2 h. After cooling to room temperature, polyethylene glycol (PEG) was combined with a hexamethylene diisocyanate (HDI) solution at a 1:2 molar ratio. This PEG‐HDI mixture was then added (10 wt%) to the DMF/TPU solution (Macklin Biochemical Technology Co., Ltd.) and stirred magnetically until completely dissolved. Subsequently, the mixed solution was irradiated under a 365 nm UV lamp for 0, 10, 20, 30, and 40 mins, yielding a modified solution. This solution was thoroughly blended with NaCl, poured into a 25 × 25 mm silicone mold, and allowed to rest for 5 min. Finally, the mold was immersed in 50 °C water for 24 h. The resulting modified porous TPU scaffold was then demolded.

### Preparation of LM Ink

A 0.05 wt% sodium alginate solution (analytical reagent, 90% purity, Macklin Biochemical Technology Co., Ltd.) was dissolved in deionized water. The solution was heated to 60 °C under stirring for 30 mins. After cooling to room temperature, 10 wt% liquid metal (LM, purchased from Huatai Materials Technology Co., Ltd.) was added dropwise. The resultant mixture was then dispersed using an ultrasonic probe operating at 20 kHz with an output power of 800 W. Ultrasonication was performed for varying durations (5, 10, 20, 30 min) to obtain LM inks. The probe utilized had a tip diameter of 4 mm and operated at an amplitude setting corresponding to 60% of its maximum power capacity.

### Characterization and Measurement

Surface morphology of materials was measured by the tungsten filament scanning electron microscopy (VEGA3 TESCAN). The contact angle of the material was tested by the contact Angle tester (SDC 350KS). Fourier transform infrared spectroscopy (SDC 350KS) was used to characterize the chemical composition of the modified TPU. Sensing performance is composed of a tensile machine (PY‐880), SR570 low noise current amplifier (Stanford Research System), Multifunctional lock‐in amplifier (Moku:Go). The in vitro antibacterial activity detection of the sensor is carried out by an electronic balance (FA2004), an electric heating constant temperature incubator (XDP‐B2160), a high‐temperature and high‐pressure sterilizer (HirayamaHVE‐50), a constant temperature shaker (IS‐RDV1), and a clean bench (SW‐CJ‐2FD). The multi‐channel signal acquisition system (04M‐V3‐Bl) with data acquisition capability of 16 channels simultaneously was used to test the sensor array. The signal acquisition system was connected with the application (App) of a mobile phone through a wireless serial port. All the biological signals involved in this paper were collected from volunteers with informed consent, and the experiments described in this paper met local ethical requirements.

## Conflict of Interest

The authors declare no conflict of interest.

## Supporting information



Supporting Information

## Data Availability

The data that support the findings of this study are available from the corresponding author upon reasonable request.
