# Supplementary material for: Strain‐Adaptive Liquid Metal Interfaces Overcome Poisson's Ratio Constraints in Piezoresistive Sensors for Infant Sleep Monitoring
Source: Adv Sci (Weinh). 2025 Sep 14;12(44):e15117. doi: 10.1002/advs.202515117 (PMC12667455; doi:10.1002/advs.202515117)
Supplement: Supplementary file 1 — Supporting Information [file ADVS-12-e15117-s001.docx]

Supporting Information

**Strain-Adaptive Liquid Metal Interfaces Overcome Poisson’s Ratio Constraints in Piezoresistive Sensors for Infant Sleep Monitoring**

*Yuxiao Zhang, Chenchen Wang, Juan Tao, Shaoyu Ma, Siyu Xie, Weiwei Guo, Rongrong Bao, Jianbei Qiu, Yue Liu*, Zhengwen Yang* and Caofeng Pan**

Y. Zhang, S. Xie, W. Guo, Prof. J. Qiu, Y. Liu, Prof. Z. Yang

College of Materials Science and Engineering, Kunming University of Science and Technology, Kunming 650093, P. R. China

Corresponding Authors’ E-mail: liuyue2023@kust.edu.cn, yangzw@kust.edu.cn

Prof. R. Bao, Prof. C. Pan

Institute of Atomic Manufacturing, Beihang University, Beijing 100191, P. R. China

International Institute for Interdisciplinary and Frontiers, Beihang University, Beijing 100191, China

Corresponding Authors’ E-mail: 11513@buaa.edu.cn (pancaofeng@buaa.edu.cn)

Z. Yang

Southwest United Graduate School, Kunming, 650093, China

J. Tao, S. Ma

Jiashan Fudan Institute, Zhejiang 314100, P. R. China

C. Wang

Henan Vocational University of Science and Technology, Henan 466000, P. R. China


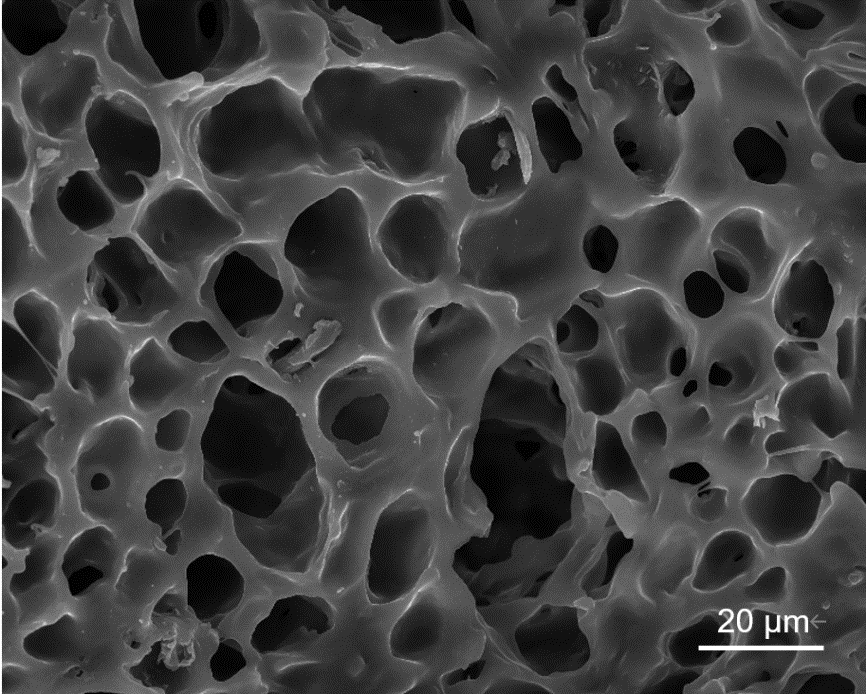


**Figure S1.** The SEM image of TPU porous scaffold. (Accelerating voltage: 20 kV)


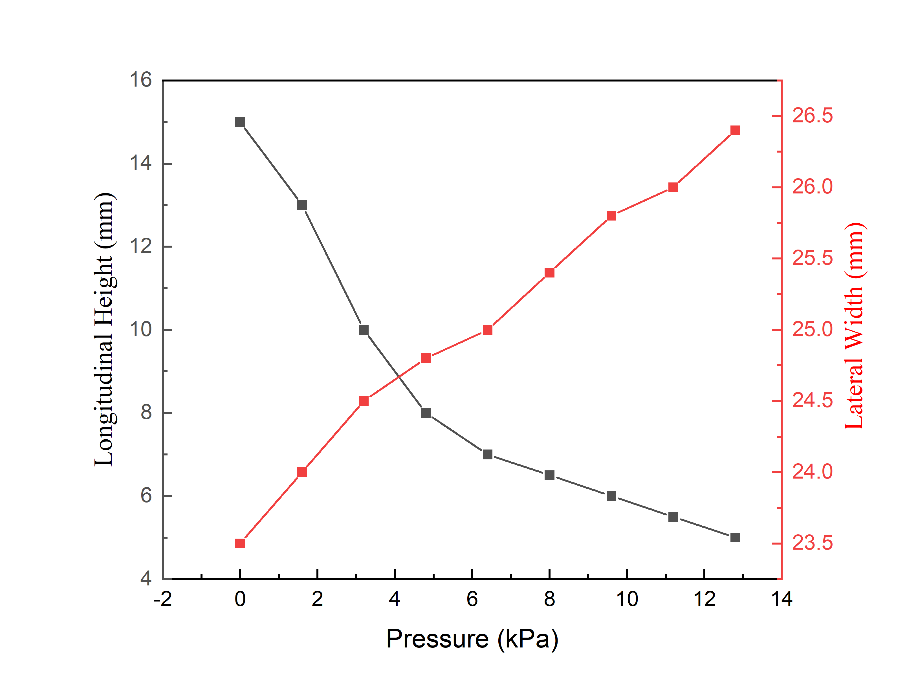


**Figure S2.** Lateral and longitudinal strain values under normal pressure.

**Equation S1.** The Poisson's ratio (*ν*) is calculated as:
​**​**

*ν*=− ​*ε*_trans_​​​​/*ε*_axial_   **(1)**

*ε*_trans​_=Δ*d*/*d*_0_

*ε*_axial​_=Δ*L*/*L*_0​_

where, *ν* (​​nu​​) is Poisson’s ratio (dimensionless), *ε*_trans_​ is ​​transverse strain​​ (strain perpendicular to applied force), Δ*d* is change in width/diameter, *d*_0_​ is original dimension), *ε*axial​ is ​​axial strain​​ (strain parallel to applied force), Δ*L* is change in length, *L*_0​_ is original length.


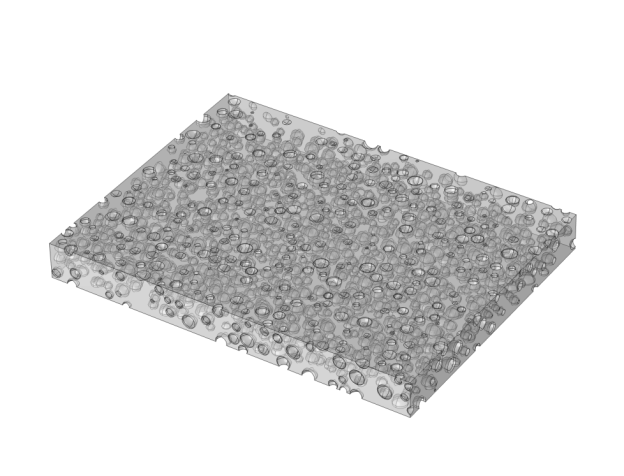

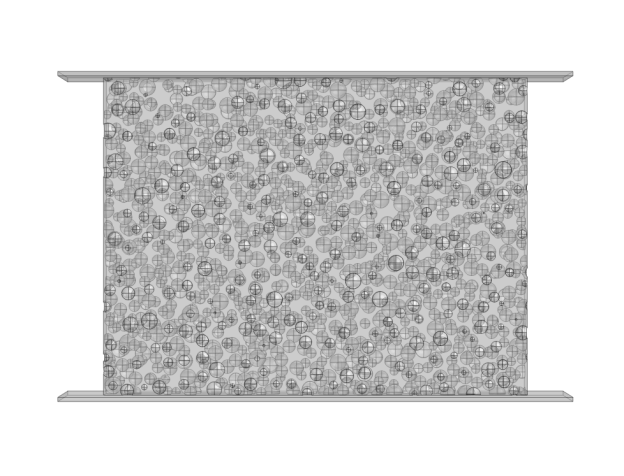


**Figure S3. Finite element simulation of porous model.​**


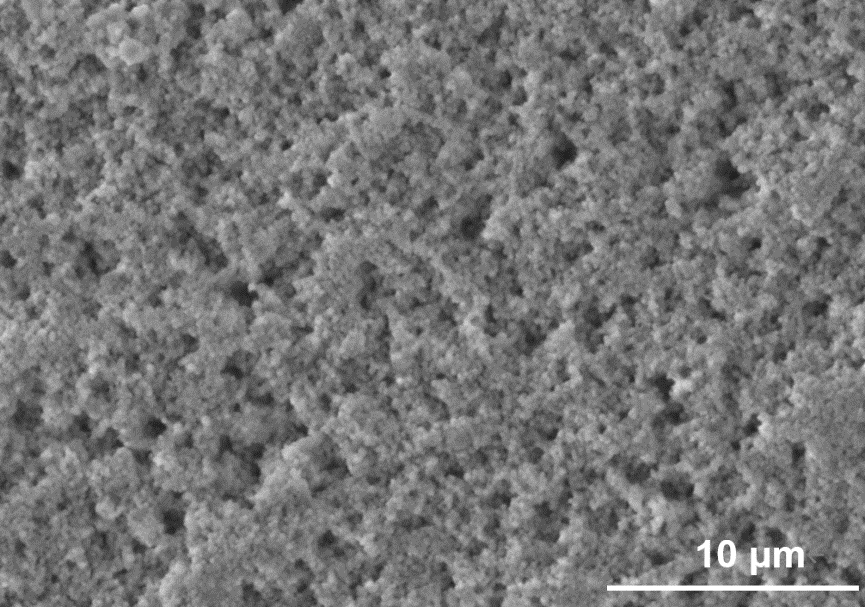


**Figure S4.** The SEM image of the LM ink after 40 minutes of sonication​. (Accelerating voltage: 10 kV)


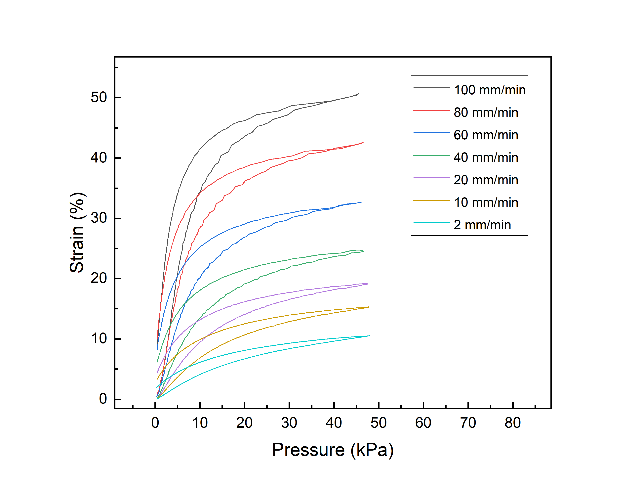


**Figure S5.** Stress-strain curves of the porous sensing layer at various strain rates.

Test results indicate that under force of the same magnitude, the faster the force is applied (at high strain rates), the greater the deformation (strain). This phenomenon is closely related to the porous structure, the viscoelasticity of TPU itself, and the behavior of gas within the pores. The core reason lies in the behavior of air inside its porous structure. When force is applied rapidly, the air in the pores cannot escape in time and undergoes adiabatic compression, causing a sharp increase in gas pressure and temperature. The high-pressure gas thus supports the TPU skeleton and significantly shares the external load, reducing the net force actually acting on the solid skeleton. As a result, the deformation of the skeleton is smaller. However, the dramatic compression of the gas volume itself contributes the vast majority of the macroscopic deformation. In contrast, during slow application of force, the gas has ample time to escape, and deformation is primarily dominated by the viscoelastic deformation of the skeleton. Under the same external force, the total deformation is actually smaller. Therefore, the larger deformation observed under high-speed loading is essentially due to the substantial reduction in volume caused by the adiabatic compression of gas within the pores. This represents a unique manifestation of the gas-solid coupling effect in porous materials, in sharp contrast to the strain rate hardening behavior exhibited by dense materials.

​
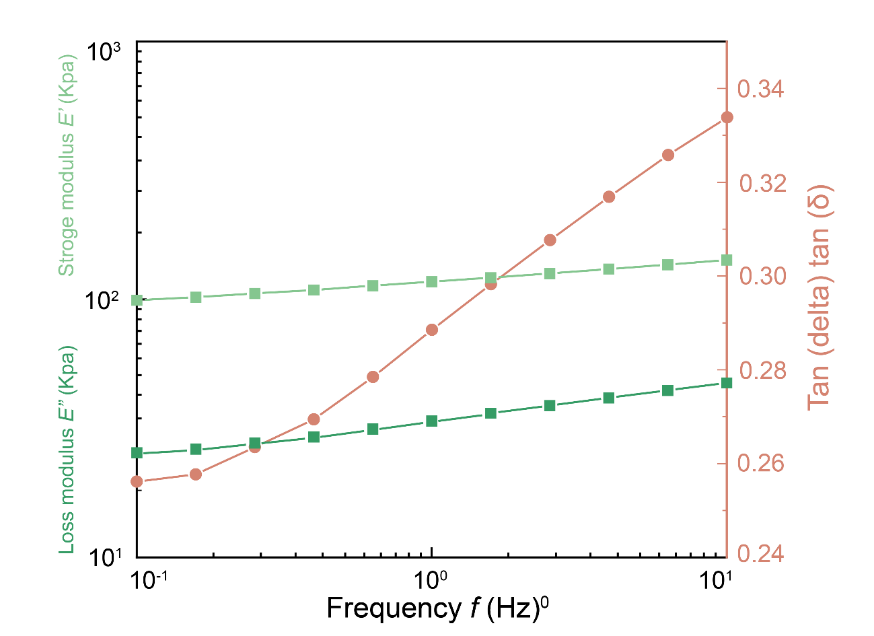


**Figure S6.** DMA data analysis plot for porous TPU film based on isothermal frequency sweep mode.​

The viscoelastic mechanical properties of the porous TPU film exhibit significant and regular frequency dependence within the tested frequency range of 0.1 Hz to 10 Hz. Under the tested room temperature conditions, the material's behavior primarily displays typical characteristics of a glassy polymer. The initial value of the storage modulus (E') at the low-frequency end (0.1 Hz) is approximately 0.01 MPa. This low value reflects the low stiffness of the porous film. As the frequency increases, E' shows a slow but persistent upward trend throughout the entire frequency range. This indicates that the relaxation time of the molecular chain segments exceeds the loading time at low frequencies, but responds more strongly to high-frequency stimuli. The loss modulus (E'') remains relatively stable and low in the low-frequency region (< ~10 Hz) (on the order of approximately 0.01 MPa), but significantly increases in the high-frequency region (> ~10 Hz). The loss factor (tan δ) exceeds the typical glassy plateau value of 0.01-0.1 across the entire frequency range, starting at an initial value of about 0.24-0.25 and monotonically rising to approximately 0.34 with frequency. tan δ > 0.1 and its increase with frequency indicate that this porous TPU film at room temperature resides in a transition region, likely dominated by a secondary transition (such as the soft segment glass transition) occurring not far below the glass transition temperature (Tg). Molecular chain segments still retain some mobility within this region, but it is insufficient for complete relaxation. The continuous rise in tanδ, combined with the significant increase in E'' in the high-frequency region, indicates that the material's internal viscous dissipation mechanisms become increasingly important with rising frequency. More energy is dissipated through friction (viscosity) between molecular chains rather than being stored purely elastically. This aligns with the time-temperature superposition behavior of materials in their transition region.


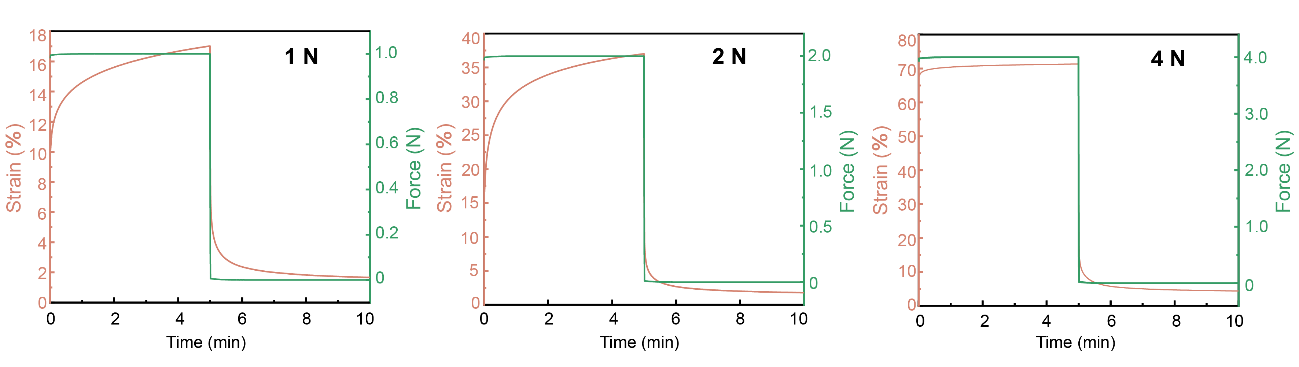


**Figure S7.** Creep and recovery curves of the porous sensitive layer under different loads (1 N, 2 N, 4 N).

Under a 1 N load, during the creep stage from 0 to 5 mins, strain gradually increases from 0% to approximately 15%, while the creep rate progressively decreases over time. This is attributed to reversible elastic deformation of TPU molecular chains under low stress, accompanied by slippage between molecular chains, leading to a slow increase in strain. After 5 mins, the load is removed, entering the recovery stage. Following stress unloading, the strain instantly rebounds by about 5%, then slowly decreases to approximately 3%, finally stabilizing. The residual strain is only 3%, with a remarkable recovery rate reaching 97%, demonstrating TPU's excellent elastic recovery performance under low loads. Under a constant 2 N load, strain ​​increases abruptly to ~35%​​ and then levels off. Compared to the 1 N load, the strain increase is larger and the time to reach steady state is shorter. The stress increase induces intensified molecular chain slippage, compression of the pore structure, and an increased contribution of plastic deformation. After 5 min, the load is removed. Upon unloading, strain decreases to ~8%, ​​resulting in a residual strain of 8%​​. The recovery rate is approximately 73%, significantly lower than under 1 N. The recovery curve exhibits a flatter slope than before, indicating that the high stress prevents partial molecular chains from fully retracting. Under a constant 4 N load, the material strain nonlinearly increases from the initial 65% to 70%. After unloading, the strain instantaneously rebounds to 5%, indicating that pore collapse is reversible. The pore walls can fully rebound via the entropic elasticity of molecular chains following buckling. Moreover, TPU hard domains reorganize under high stress to form a stable support network. The vertical drop and plateau stabilization demonstrate the absence of viscous flow hysteresis. Compared to low-load behavior, residual strain under high stress is lower, with a lower proportion of energy dissipation.


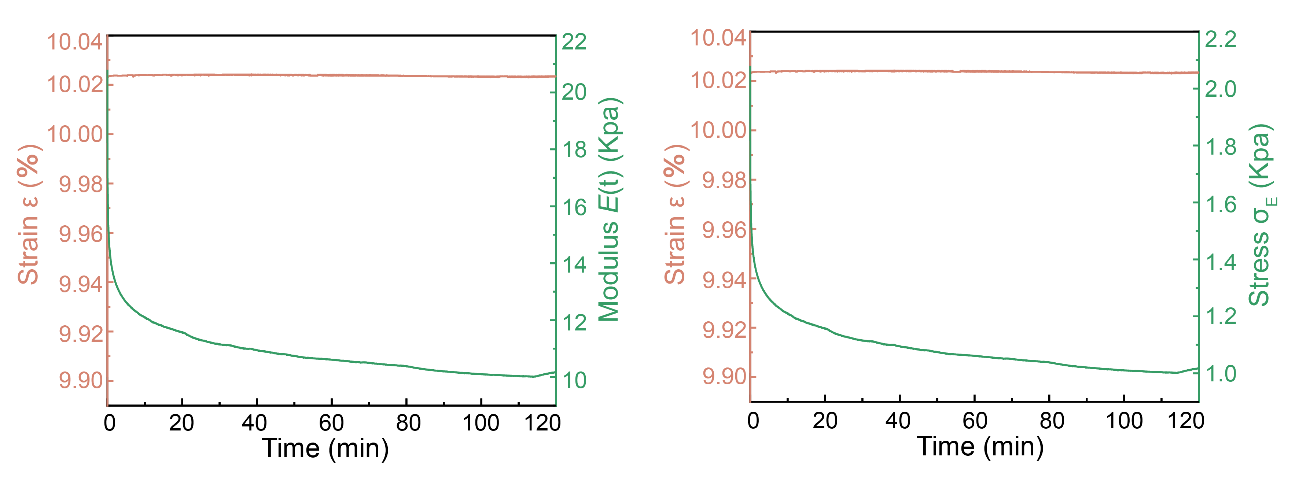


**Figure S8.** Stress relaxation profile of the porous sensing layer during a 120-minute period.​

This stress relaxation curve illustrates that the porous TPU membrane is a typical viscoelastic solid, its stiffness possessing significant time dependence (relaxation). The stress required to maintain a constant strain over 120 mins decreased by approximately 28.6%. The rapid relaxation segment of the material occurs within the first few tens of minutes. The stress decreased very rapidly initially, starting from 0.014 MPa. At 20 min, the stress had already dropped to ~0.0108 MPa, a decrease of about 23%. This indicates rapid internal structural adjustments within the material to adapt to the applied strain. Subsequently, the stress decrease rate significantly slowed, in the subsequent 100 mins, from ~0.0108 MPa dropping to 0.010 MPa, a decrease of only ~7.4%. The curve at the 120-minute mark is not completely horizontal, meaning ​​relaxation has not yet ended​​. Stress might continue to decrease slowly. The fast then slow behavior is due to ​​polymer viscoelasticity​​. The initial decay corresponds to the rapid motion and rearrangement of polymer chain segments and small molecules. The slow decay in the mid-to-late stage corresponds to slower molecular processes. Additionally, the ​​presence of pores reduces​​ the material's overall load-bearing cross-sectional area and stiffness. The initial stress causes changes in the pore structure. ​​Stress relaxation is related to​​ the hysteresis effect, meaning the porous TPU membrane can absorb and ​​dissipate energy​​ during dynamic loading-unloading cycles. The rapid initial stress relaxation helps quickly ​​dissipate impact energy​​. However, ​​if a device needs to​​ bear a constant deformation for a long time, the initial pre-load/strain needs to be corrected based on the stress expected after the intended usage time. In this experiment, the initial pre-load has been corrected.


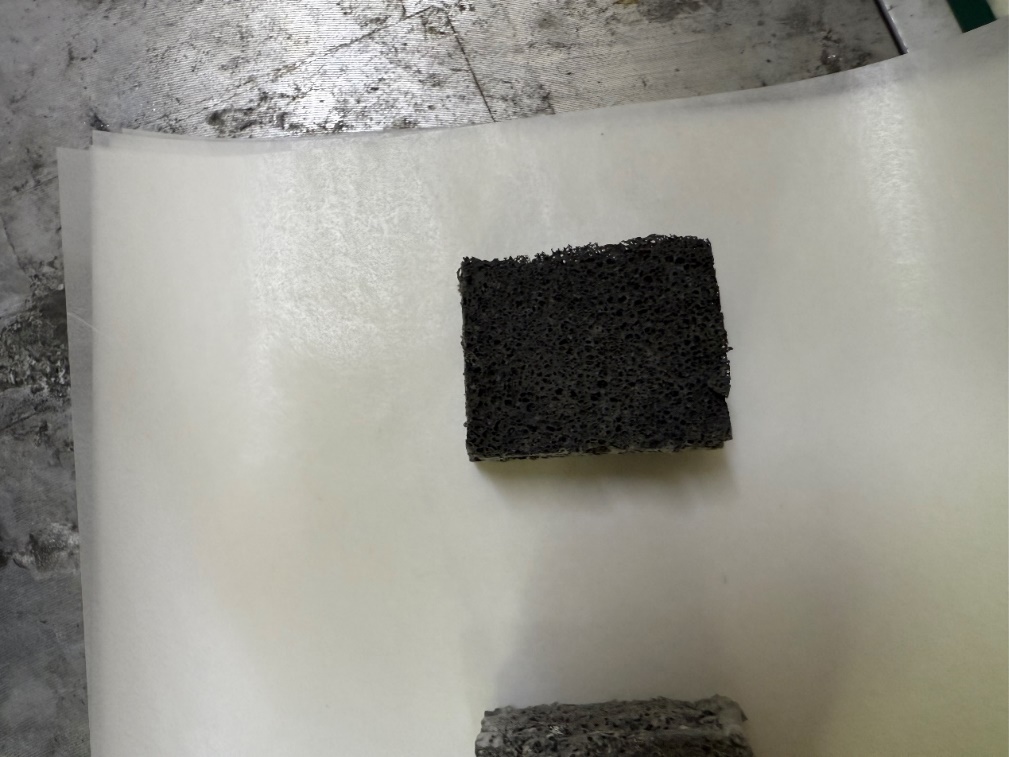

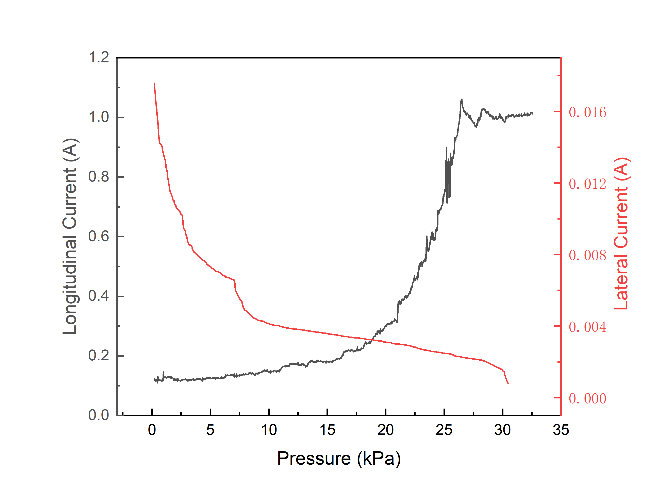


**Figure S9.** Device based on graphite powder and porous TPU and its lateral and longitudinal current variations under pressure.​

A porous TPU scaffold coated with ​​graphite powder​​ (40 wt%) was subjected to ​​normal pressure (0.2-32.57 kPa) via an integrated strain-conductivity characterization system, with ​​real-time resistance monitoring​​ in both transverse and longitudinal orientations. The ​​consistently observed conductivity enhancement under longitudinal compression​​ (＞700% increase at 32.57 kPa) and ​​suppression under transverse tension​​ (95% decrease at 30.47 kPa) fully corroborate the theoretical predictions.


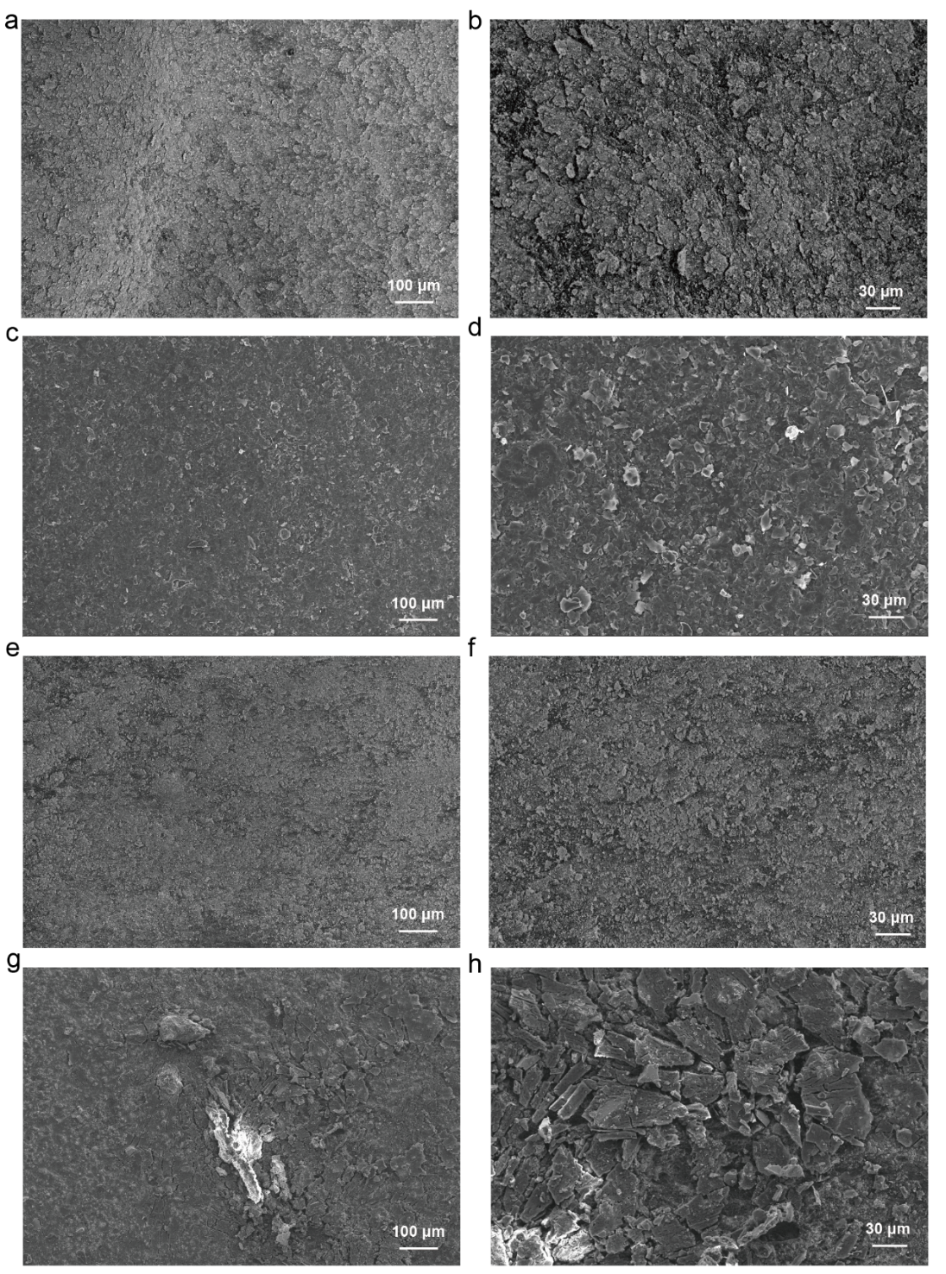


**Figure S10.** The interconnectivity status of this liquid metal ink versus conventional rigid material graphite powder under applied stress (Accelerating voltage: 10 kV).

To demonstrate the gap-filling capability of liquid metal enabled by its fluidity during tensile strain, the film embedded with graphite particles was used as the sensing material for the control group and subjected to SEM testing. Figure S10a-d depict the status of the sensing materials on the TPU film prior to tensile testing. Specifically, Figures a and b show liquid metal at distinct magnification levels, while Figure S10c and S10d present graphite powder at varying magnifications. Although both sensing materials exhibited relatively flat surfaces, the liquid metal ink demonstrated relatively weaker connectivity when not subjected to stress activation due to its higher surface tension. As a comparison, Figure S10e-h present SEM images of the two films under 20% tensile strain. The results demonstrate that distinct macroscopic cracks formed in the graphite powder after 20% tensile strain. In contrast, no obvious cracks were observed in the liquid metal morphology. Instead, its connectivity became better than in the unstretched state. Based on published literature, this phenomenon may be attributed to the rupture of the surface oxide layer on the liquid metal ink under stress, causing the internal liquid metal to flow out. Through comparative experiments with conventional rigid graphite powder particles, it can be proven that this liquid metal ink avoids the formation of large cracks after stretching due to its fluidity.


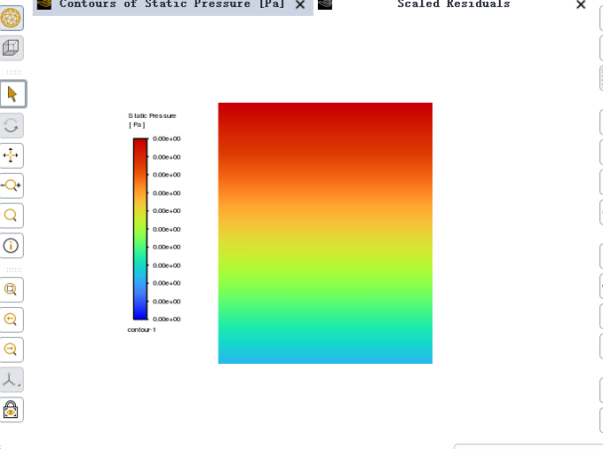

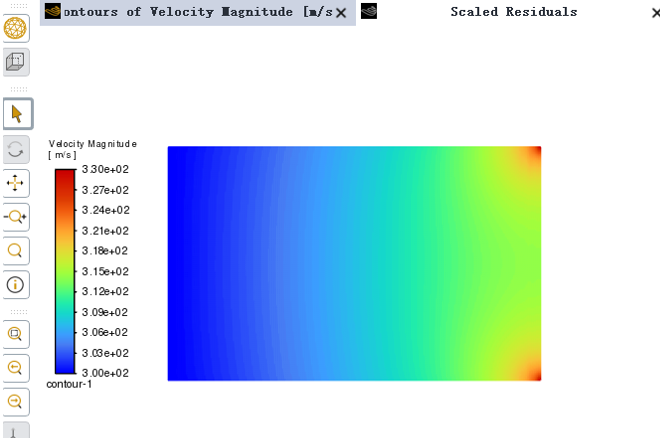


**Figure S11.** Simulation of the strain-flow coupling process in LM.


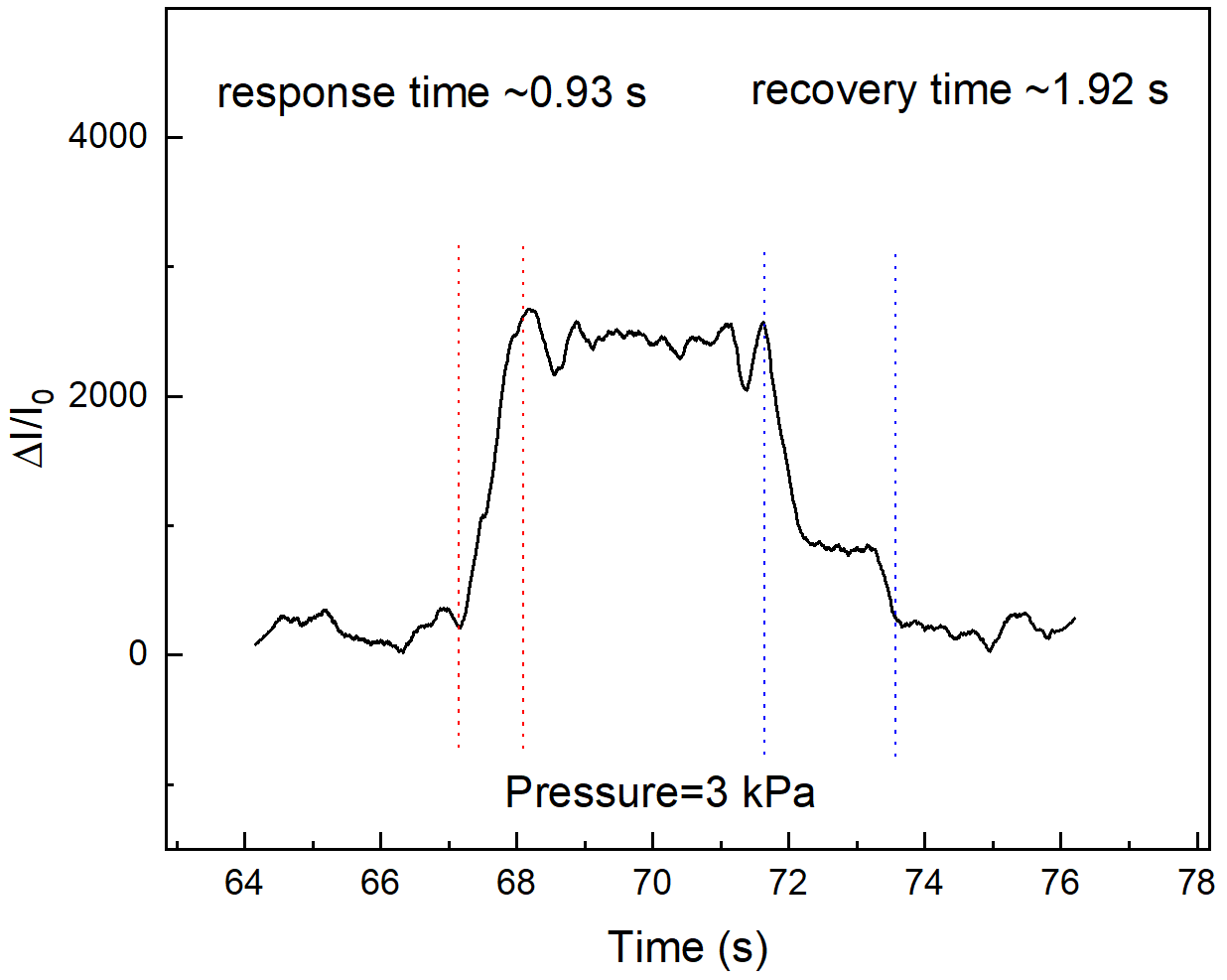


**Figure S12.** The response time and recovery time of the pressure sensor.


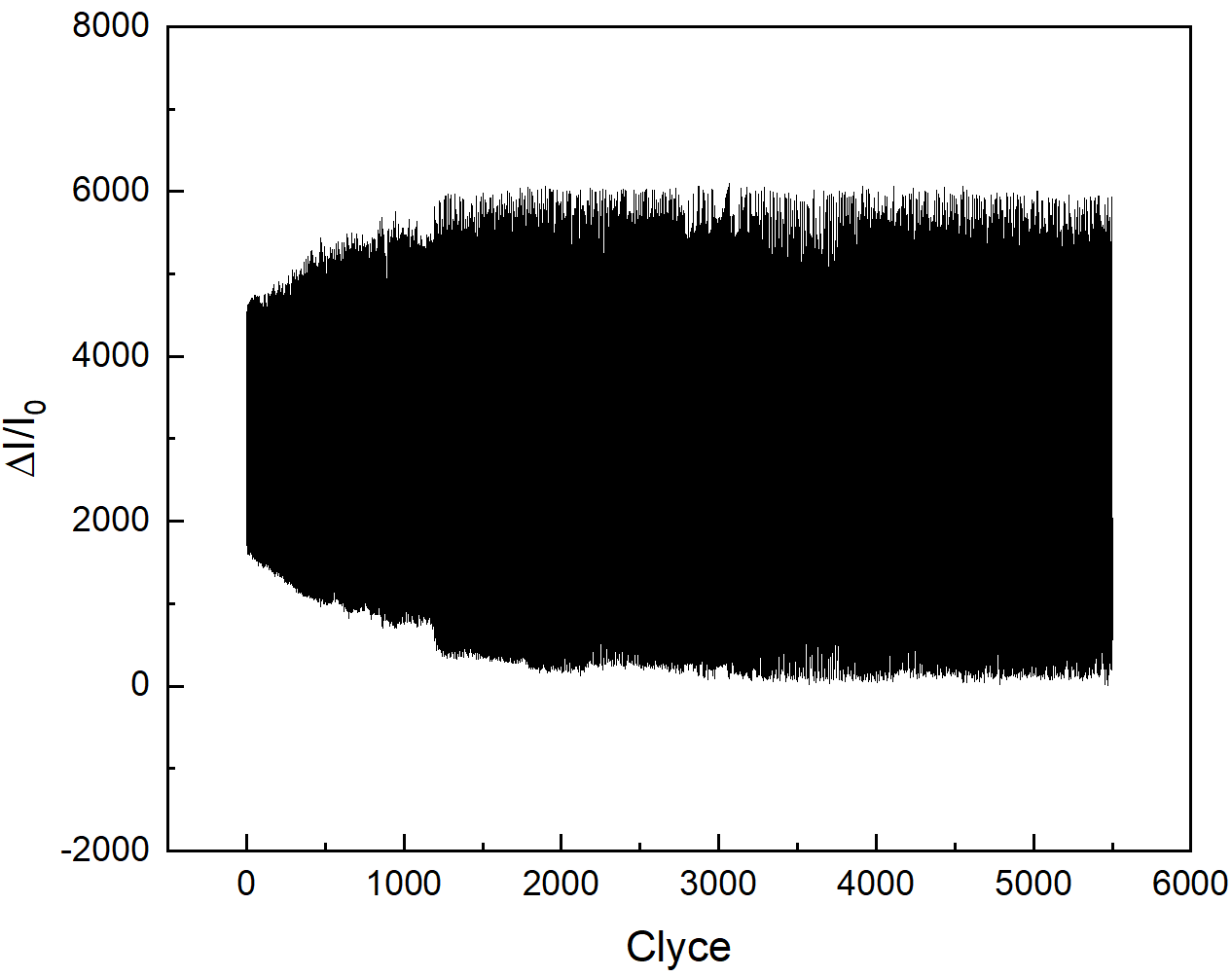


**Figure S13.** The compression cycles test of the pressure sensor.


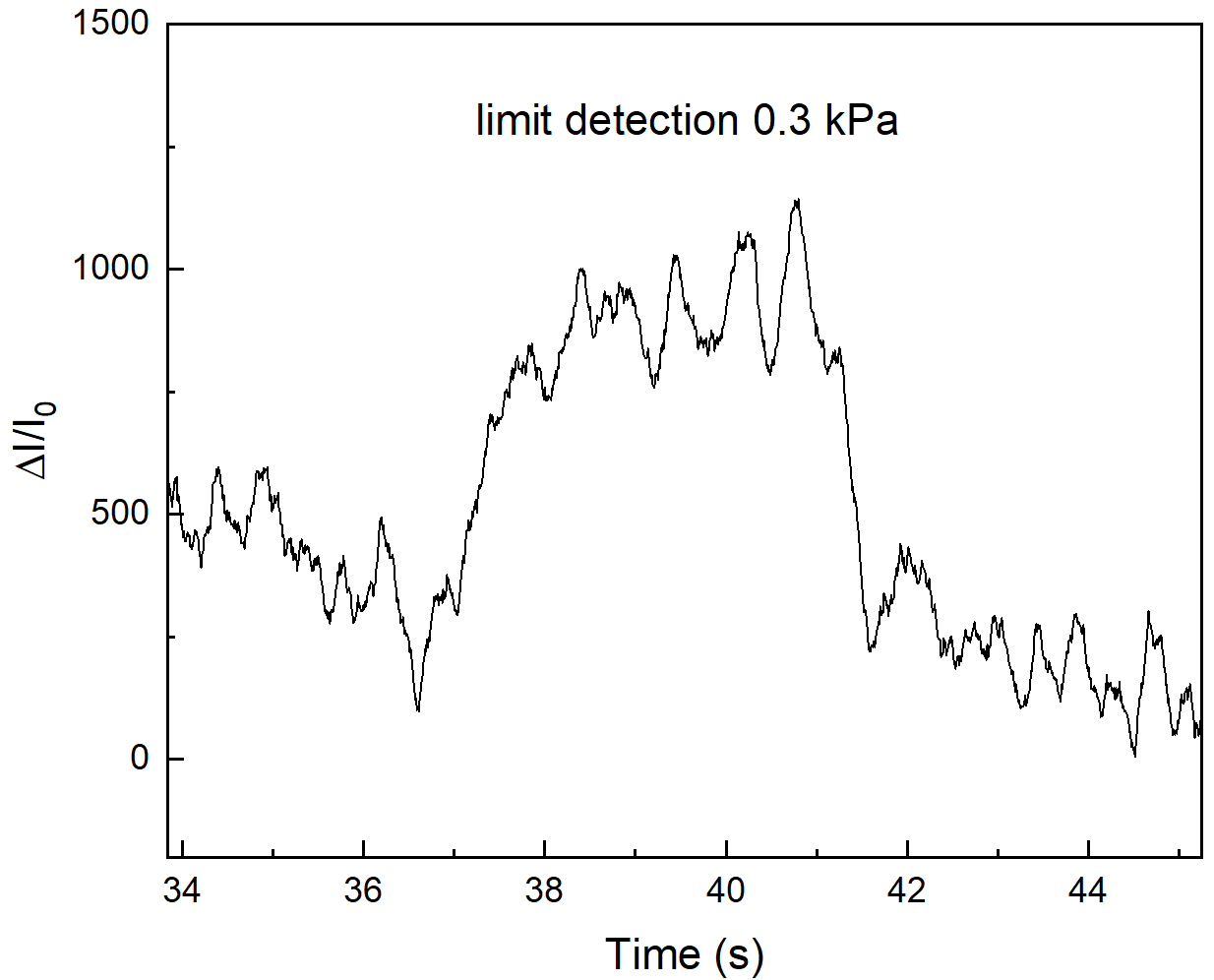


**Figure S14.** The minimum detection limit of the pressure sensor.


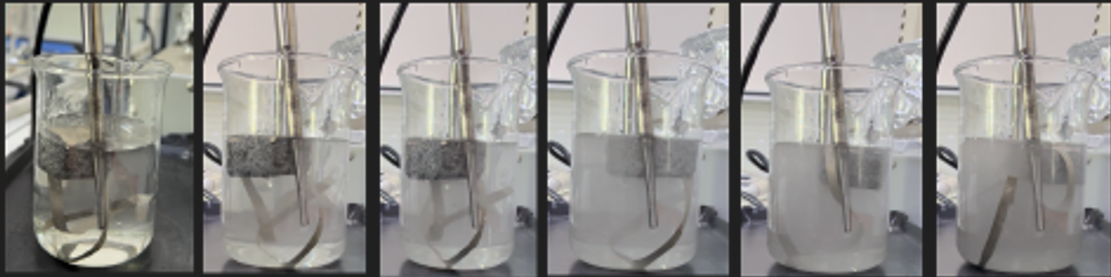


**Figure S15.** The launderability test of the sensor.


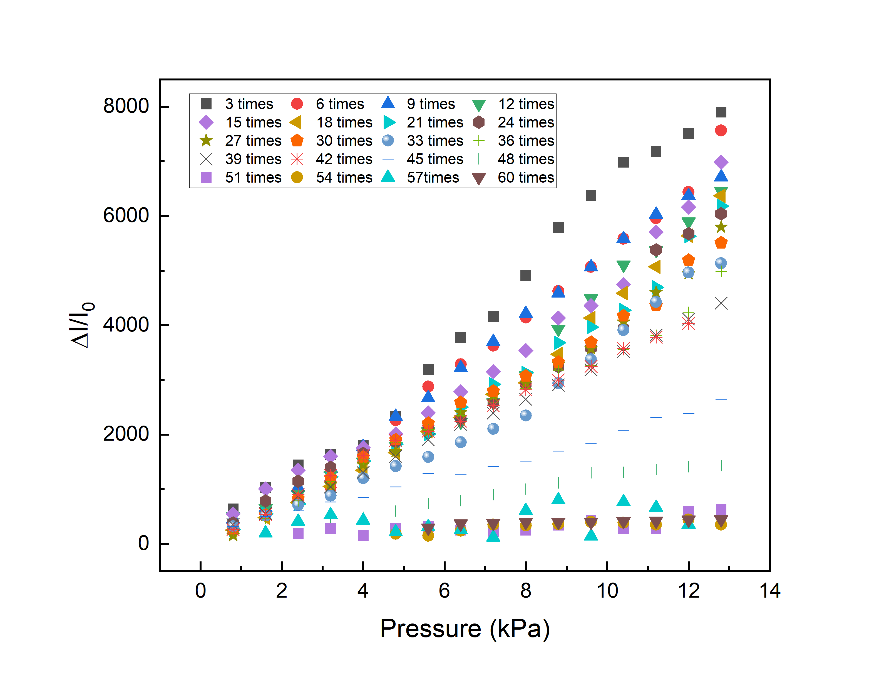


**Figure S16.** Validation was performed with 60 wash cycles using a traditional washing machine.

Experimental validation confirms that the device maintains ​​49.8% (The initial sensitivity is 660.51 kPa^-1^. After 42 wash cycles, it now measures 328.94 kPa^-1^.​) pressure response sensitivity​​ after 42 cycles of ​​realistic cleaning protocols​​. Given the ​​quarterly laundering schedule​​ established for infant care products, this durability corresponds to ​​over 10 years of equivalent service life​​ under normal operating conditions. The table of wash cycles versus sensor sensitivity is shown below.

| Number of Wash Cycles​ | Sensitivity (kPa^-1^) | Retained Value​​ (%) |
| --- | --- | --- |
| 3 | 660.51 | 100 |
| 6 | 595.97 | 90.23 |
| 9 | 552.51 | 83.65 |
| 12 | 507.99 | 76.91 |
| 15 | 500.94 | 75.84 |
| 18 | 495.77 | 75.05 |
| 21 | 471.03 | 71.31 |
| 24 | 443.02 | 67.01 |
| 27 | 434.43 | 65.77 |
| 30 | 421.61 | 63.83 |
| 33 | 411.13 | 62.24 |
| 36 | 362.45 | 54.87 |
| 39 | 337.79 | 51.14 |
| 42 | 328.94 | 49.80 |
| 45 | 185.79 | 28.13 |
| 48 | 113.32 | 17.16 |
| 51 | 30.53 | 4.62 |
| 54 | 28.51 | 4.32 |
| 57 | 23.54 | 3.56 |
| 60 | 16.28 | 2.46 |

**Table S1.** The number of wash cycles, sensitivity, and retained value of the device in traditional washing machines.


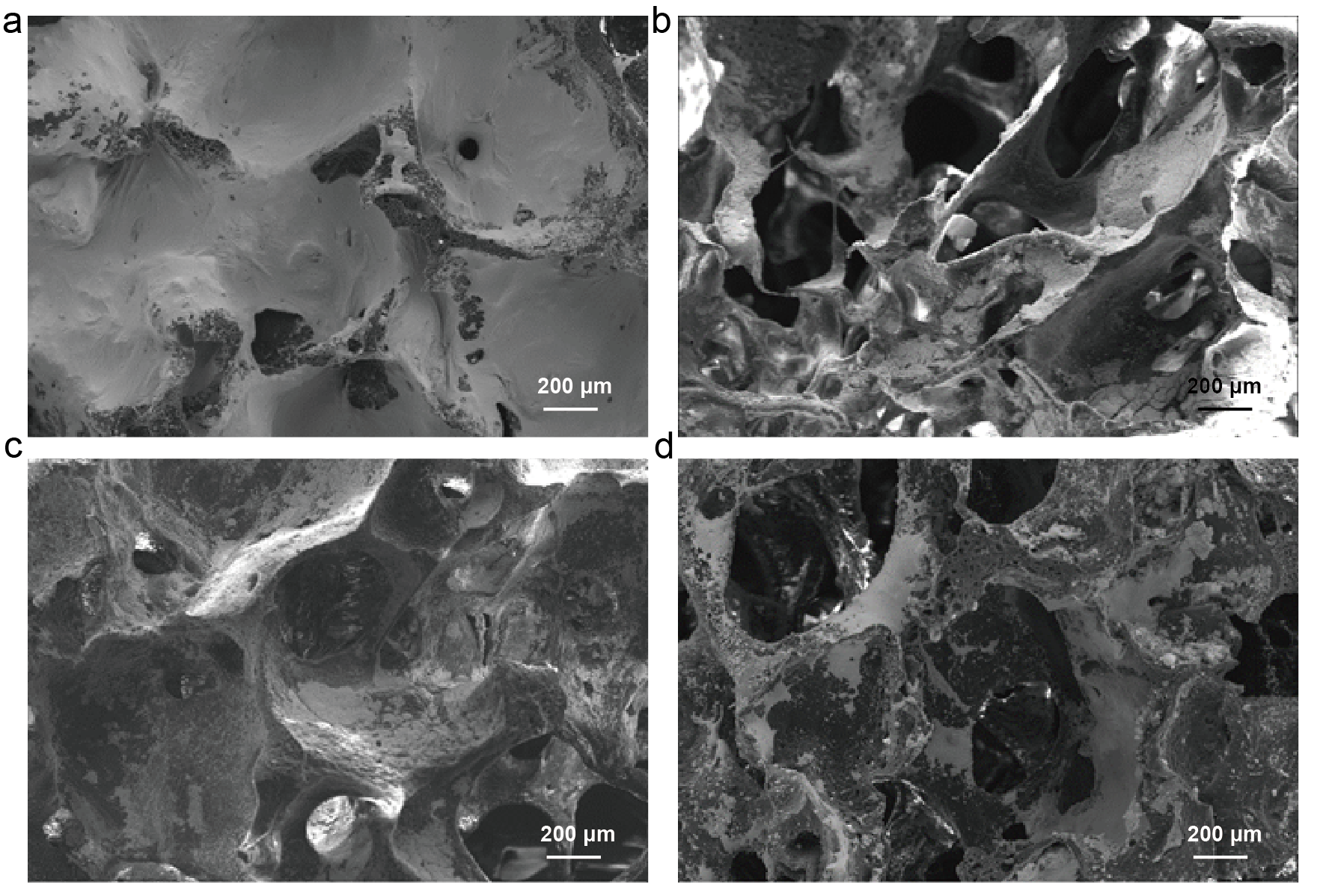


**Figure S17.** Adhesion levels of liquid metal on TPU substrate under varying washing cycles (0, 21, 42, 60 cycles, each cycle lasts for 25 seconds) (Accelerating voltage: 15 kV).

Figure S8a-d respectively show the SEM characterization results of flexible sensing devices after 0, 21, 42, and 60 standard washing cycles (each cycle lasts for 25 seconds) using a conventional washing machine (Panasonic, rated frequency: 50 Hz, washing power: 530 W). The figures reveal that under hydrodynamic shear stress during repeated washing, functional materials on the device surface undergo progressive delamination. This reduces the sensing layer coverage from an initial 92.61% to <36.49% after 60 cycles, exposing the substrate layer. (The material coverage ratio was estimated roughly via Image J software.) As washing cycles increase, the coverage of sensing materials on the skeleton decreases. Under identical pressure conditions, ​​a decrease in the density of conductive pathways formed within the device will disrupt the percolation threshold.​ Consequently, the increase in current under pressure is limited, reducing the detectable saturation pressure. After 60 washing cycles, the sensing material coverage falls below 36.49%, and testing confirms complete device failure.


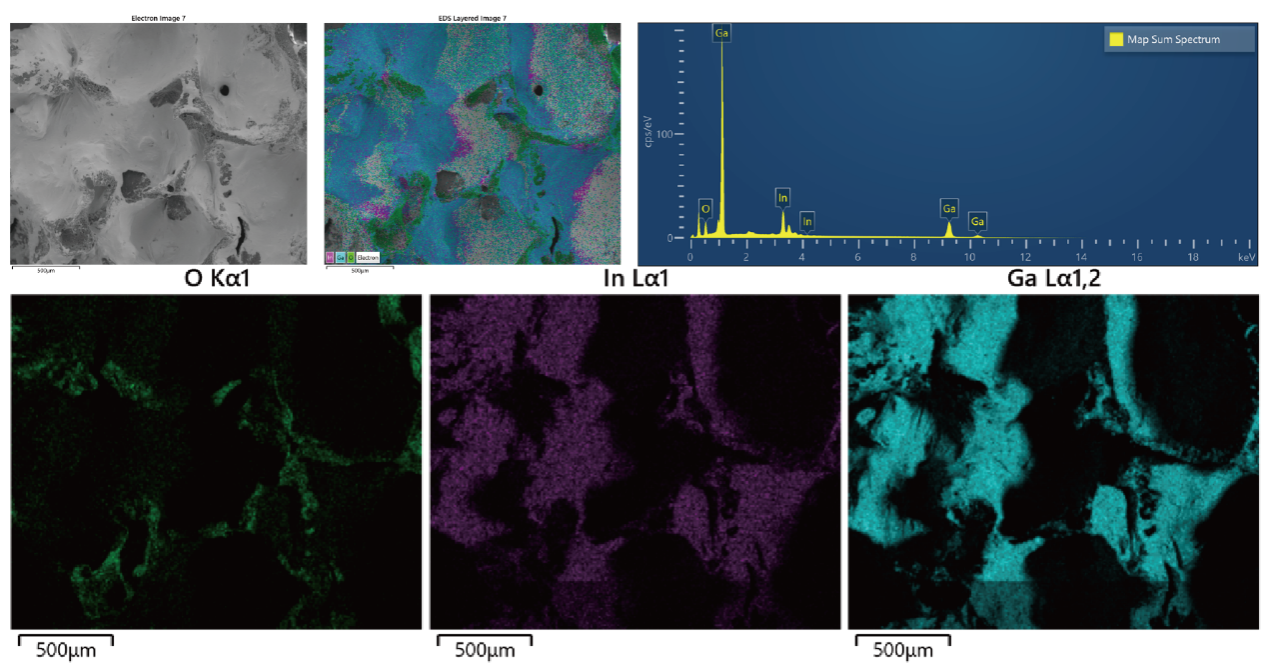


**Figure S18.** The EDS spectrum of the device when unwashed (Accelerating voltage: 15 kV).


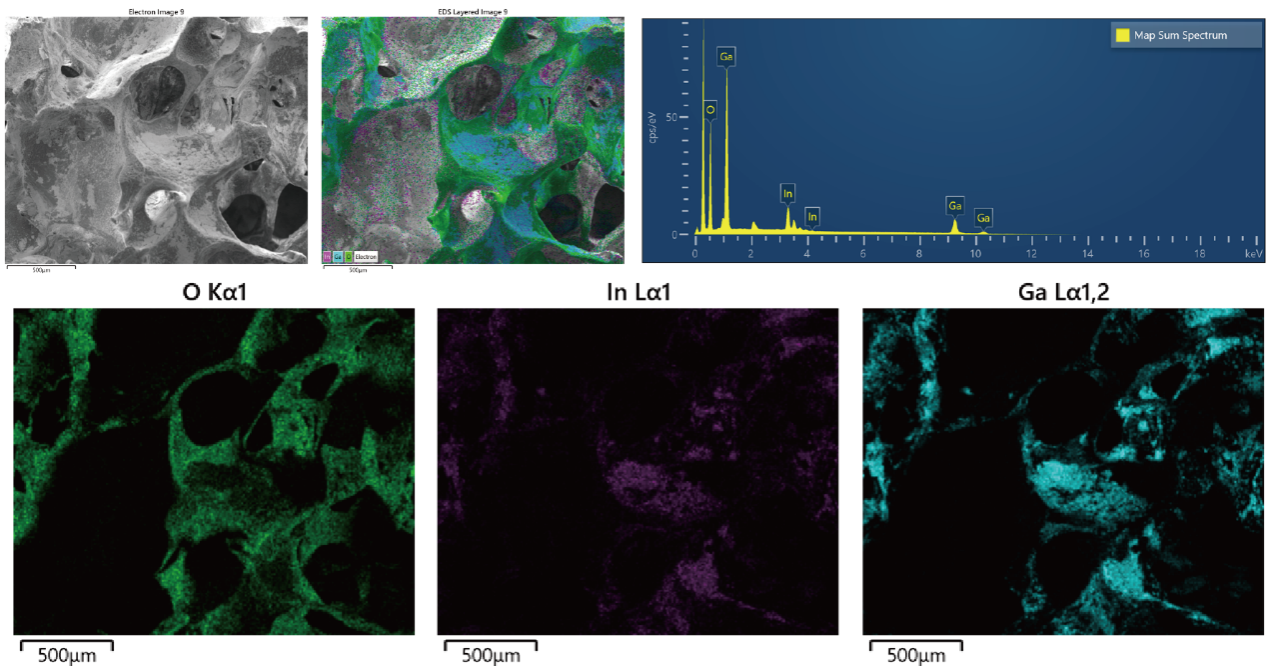


**Figure S19. ​​**The EDS spectrum of the device after 42 washing cycles (Accelerating voltage: 15 kV).​

To obtain significant contrast within the effective service life of the device, the device surfaces after 0 and 42 washing cycles have been selected for EDS analysis. The results show that when unwashed, the surface oxygen content was low, indicating a relatively low degree of oxidation of the sensing material, which favors the realization of its pressure-induced contact response. After 42 washes, EDS detected a significant increase in the proportion of oxygen, indicating a marked increase in the surface oxidation of the liquid metal. Being insulating, the thickening of the oxide layer inevitably has detrimental effects on the device's conductivity and sensing performance. However, applying a certain external pressure can rupture the surface oxide layer, allowing the internal liquid metal to flow out and reestablish conduction pathways. Therefore, although the sensing performance of the device declines with increasing washing cycles, it does not completely fail. In summary, the heightened surface oxidation of the liquid metal is one factor leading to the degradation of the device's sensing performance.


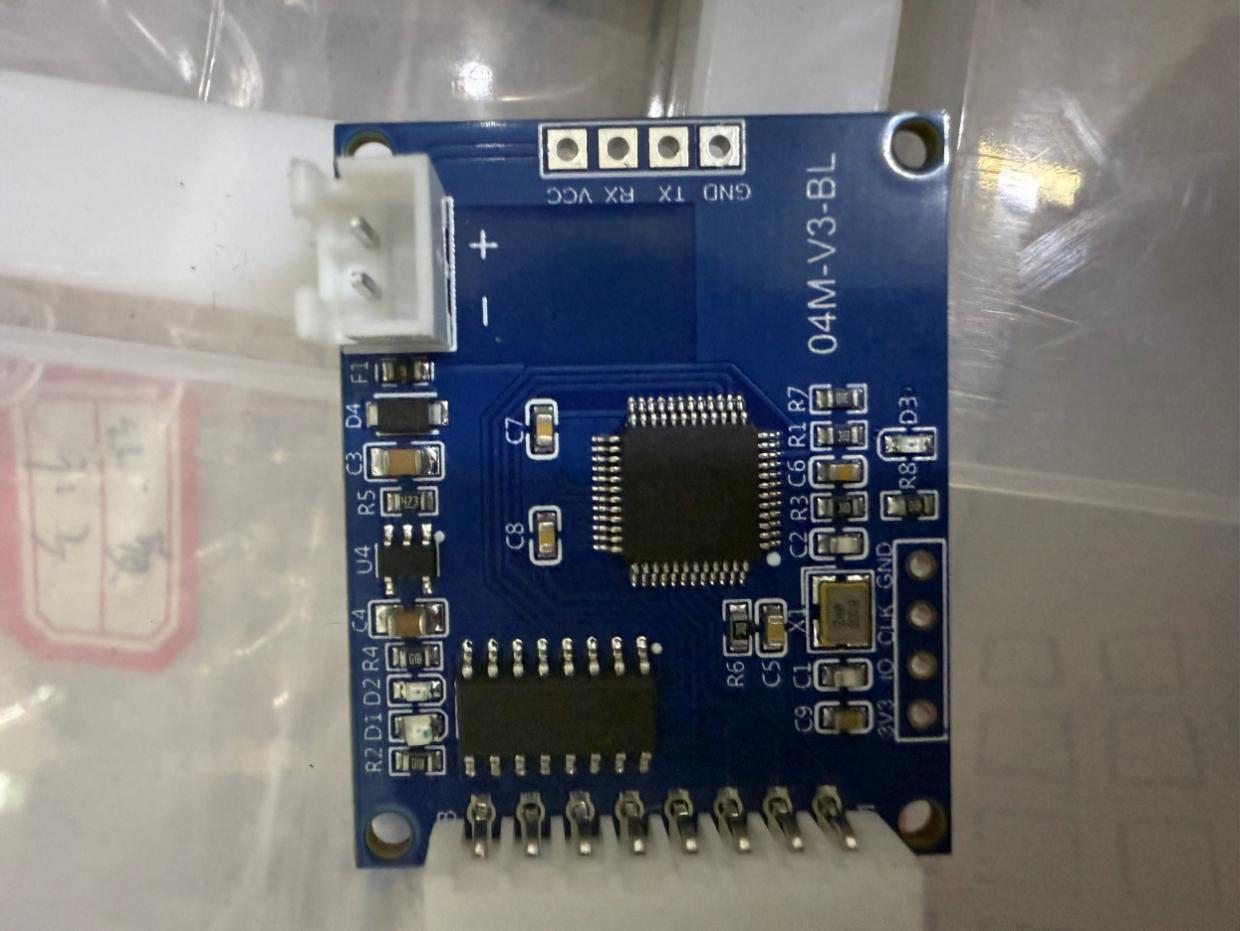


**Figure S20.** The ​chip for signal amplification and ADC conversion.


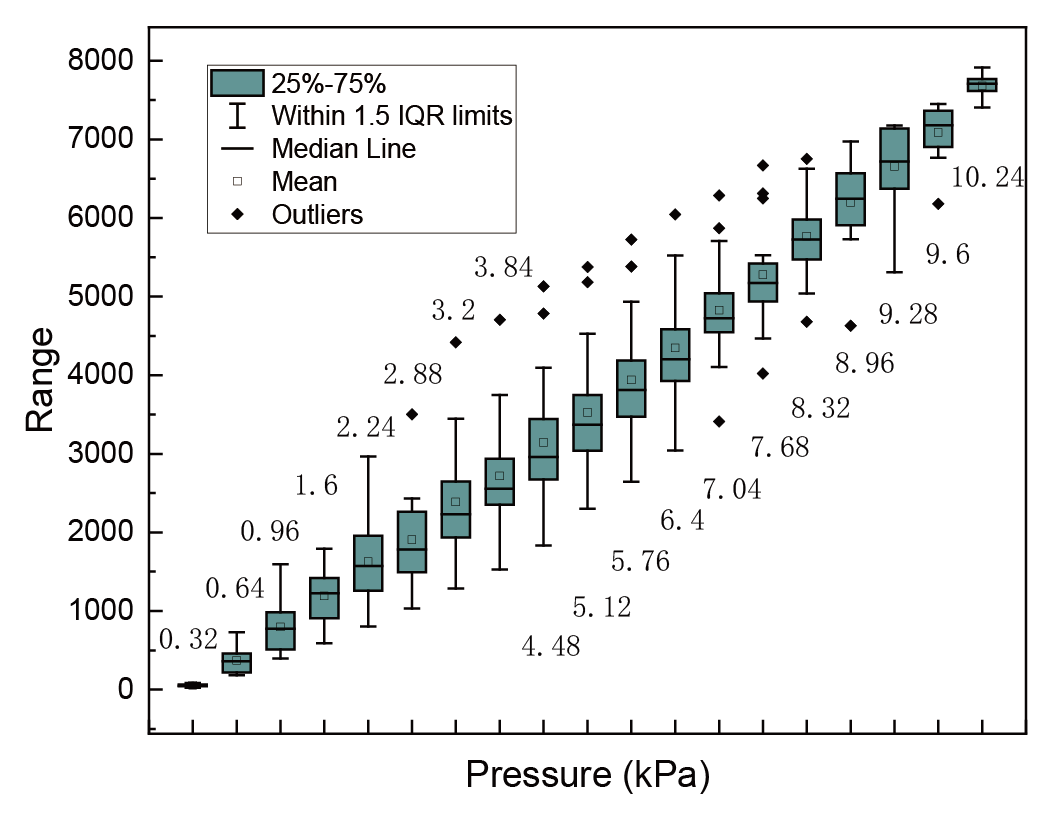


**Figure S21.** Boxplots for the 16-channel sensor array.​
